# Absolute Quantitation of Met Using Mass Spectrometry for Clinical Application: Assay Precision, Stability, and Correlation with *MET* Gene Amplification in FFPE Tumor Tissue

**DOI:** 10.1371/journal.pone.0100586

**Published:** 2014-07-01

**Authors:** Daniel V. T. Catenacci, Wei-Li Liao, Sheeno Thyparambil, Les Henderson, Peng Xu, Lei Zhao, Brittany Rambo, John Hart, Shu-Yuan Xiao, Kathleen Bengali, Jamar Uzzell, Marlene Darfler, David B. Krizman, Fabiola Cecchi, Donald P. Bottaro, Theodore Karrison, Timothy D. Veenstra, Todd Hembrough, Jon Burrows

**Affiliations:** 1 Department of Medicine, Section of Hematology & Oncology, University of Chicago, Chicago, Illinois, United States of America; 2 OncoPlex Diagnostics Inc., Rockville, Maryland, United States of America; 3 Department of Pathology, University of Chicago, Chicago, Illinois, United States of America; 4 Urologic Oncology Branch, National Cancer Institute, National Institutes of Health, Bethesda, Maryland, United States of America; 5 Department of Health Studies, University of Chicago, Chicago, Illinois, United States of America; Centro di Riferimento Oncologico, IRCCS National Cancer Institute, Italy

## Abstract

**Background:**

Overexpression of Met tyrosine kinase receptor is associated with poor prognosis. Overexpression, and particularly *MET* amplification, are predictive of response to Met-specific therapy in preclinical models. Immunohistochemistry (IHC) of formalin-fixed paraffin-embedded (FFPE) tissues is currently used to select for ‘high Met’ expressing tumors for Met inhibitor trials. IHC suffers from antibody non-specificity, lack of quantitative resolution, and, when quantifying multiple proteins, inefficient use of scarce tissue.

**Methods:**

After describing the development of the Liquid-Tissue-Selected Reaction Monitoring-mass spectrometry (LT-SRM-MS) Met assay, we evaluated the expression level of Met in 130 FFPE gastroesophageal cancer (GEC) tissues. We assessed the correlation of SRM Met expression to IHC and mean *MET* gene copy number (GCN)/nucleus or *MET/CEP7 ratio* by fluorescence in situ hybridization (FISH).

**Results:**

Proteomic mapping of recombinant Met identified ^418^TEFTTALQR^426^ as the optimal SRM peptide. Limits of detection (LOD) and quantitation (LOQ) for this peptide were 150 and 200 amol/µg tumor protein, respectively. The assay demonstrated excellent precision and temporal stability of measurements in serial sections analyzed one year apart. Expression levels of 130 GEC tissues ranged (<150 amol/µg to 4669.5 amol/µg. High correlation was observed between SRM Met expression and both *MET* GCN and *MET/CEP7* ratio as determined by FISH (n = 30; R^2^ = 0.898). IHC did not correlate well with SRM (n = 44; R^2^ = 0.537) nor FISH GCN (n = 31; R^2^ = 0.509). A Met SRM level of ≥1500 amol/µg was 100% sensitive (95% CI 0.69–1) and 100% specific (95% CI 0.92–1) for *MET* amplification.

**Conclusions:**

The Met SRM assay measured the absolute Met levels in clinical tissues with high precision. Compared to IHC, SRM provided a quantitative and linear measurement of Met expression, reliably distinguishing between non-amplified and amplified *MET* tumors. These results demonstrate a novel clinical tool for efficient tumor expression profiling, potentially leading to better informed therapeutic decisions for patients with GEC.

## Background

Hepatocyte growth factor receptor (HGFR), commonly known as Met, is a membrane receptor that possesses tyrosine kinase activity [1], [2]. Binding of HGF ligand to Met activates its kinase activity through autophosphorylation of tyrosine residues 1234 and 1235. This activation of Met engages a number of additional signal proteins (e.g., CREB, ERK1, ERK1/2, ERK2, JNK, STAT3, and various MAPKK) either directly or indirectly, resulting in a variety of Met-driven biological activities that ultimately convey an invasive oncogenic phenotype [3]. Clinically, Met is of wide-spread interest, as overexpression of this protein is associated with aggressive tumor properties and poor patient outcomes [4]–[9]. Met signaling is aberrantly constitutively activated by protein overexpression and/or genetic alteration [10]. Specifically, *MET* gene amplification and consequent overexpression is an ‘oncogenic driver’ in a subset (∼5%) of gastric and esophageal adenocarcinomas [4], [11]–[14], while *MET* mutations have been rarely reported in various hereditary and sporadic cancers including gastroesophageal adenocarcinomas (GEC) [15]. A previous study has shown that Met protein is overexpressed in esophageal adenocarcinoma (EA) surgical specimens and EA cell lines, while Met dysregulation can occur early in the progression from Barrett’s dysplasia to adenocarcinoma [16], [17]. The role of Met in GEC and other cancers have made it a prime target for therapeutic strategies [4], [14], [18]. HGF or Met inhibitors currently under development can be broadly subdivided into biological or low molecular weight synthetic compounds, and are currently being tested in clinical trials [14], [19]–[23]. Biological agents are monoclonal antibodies (mAb) that either neutralize the ligand, hepatocyte growth factor receptor (HGF), or bind the receptor itself, effectively blocking the ligand/receptor interaction and activation. These are currently being evaluated in phase I–III trials for various tumor types [18], [20], [24], [25]. In a phase I trial, we described a complete response to onartuzumab, a Met monoclonal antibody, in a patient with stage IV GEC having high *MET* GCN and Met over-expression [18]. A recent randomized phase II trial in GEC evaluating an anti-HGF antibody, rilotumumab, demonstrated a survival advantage compared to placebo, with predictive benefit particularly in patients’ tumors having high Met expression (Met+) by immunohistochemistry (IHC), in contrast to those lacking expression (Met−) [20]. On the other hand, most synthetic compounds targeted against Met are ATP competitive tyrosine kinase inhibitors (TKI) that inhibit Met autophosphorylation and subsequent downstream signaling activation, with particular sensitivity observed in the setting of Met overexpression as a consequence of *MET* amplification [13], [14], [22], [26], [27]. Notably, a single arm phase IIa trial of foretenib, a multi-kinase inhibitor including Met, was relatively disappointing, at least as monotherapy in biomarker unselected chemo-refractory GEC patients [23].

IHC analysis of formalin-fixed paraffin-embedded (FFPE) tissue sections is routinely used for measuring Met expression in patient samples. While IHC has proven useful over the past several decades, mass spectrometry (MS) methods that measure the absolute levels of proteins with high specificity for clinical application are rapidly emerging [28]. Selected reaction monitoring (SRM)-MS assays are presently widely utilized to objectively quantitate metabolites in biological samples. In contrast to IHC which has limitations in specificity, reproducibility and sensitivity, the measurements provided by SRM-MS are highly specific since several performance characteristics of the analyte are measured [29]. These characteristics include its mass, the masses of several of its fragment (or transition) ions, chromatographic retention time, as well as how well these characteristics match to a heavy isotope labeled internal standard of the analyte. Addition of the heavy internal standard also allows for absolute abundance of the target analyte to be measured.

**Figure 1:**
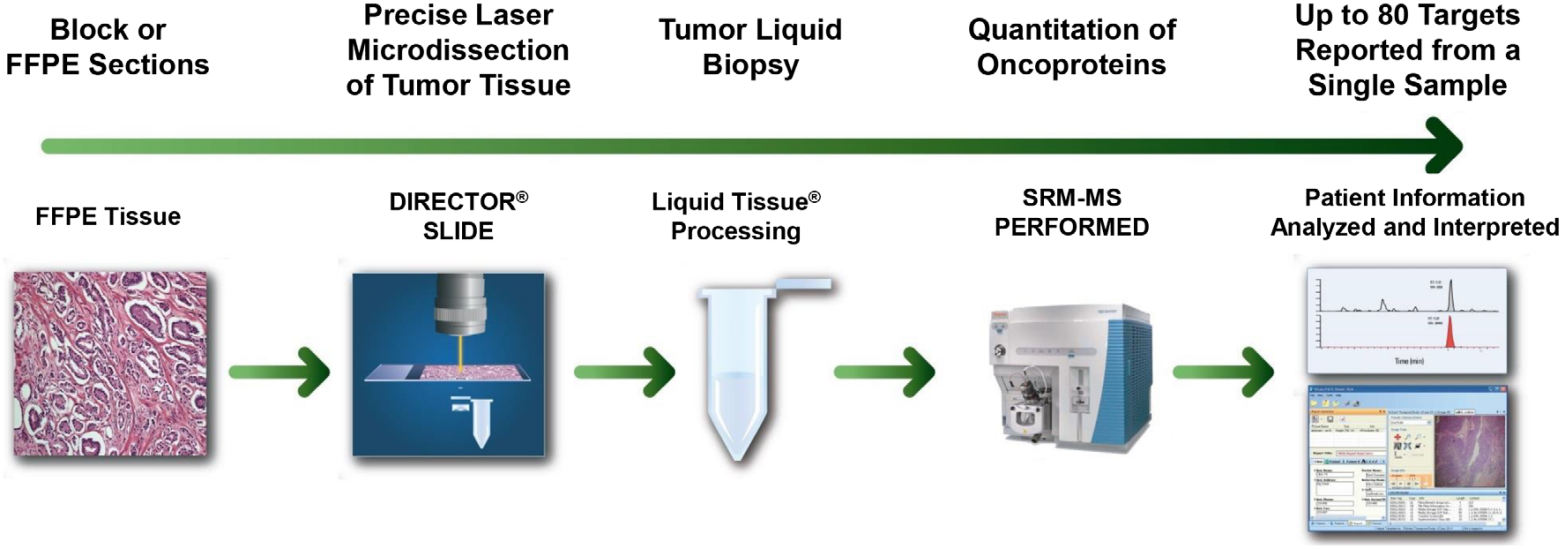
Liquid Tissue-Selected Reaction Monitoring (SRM) workflow for quantification of proteins from formalin-fixed paraffin-embedded (FFPE) tissue. Tissue sections are cut onto Director microdissection slides. After they are deparaffinized, areas of interest are identified by pathologists. Laser microdissection is used to procure areas of interest and the targeted cellular material is collected in a tube. The cellular material is processed using the Liquid Tissue protocol, which includes trypsinization, to produce a soluble peptide lysate. A known amount of heavy internal standard peptide is added to the lysate and the sample is analyzed using SRM to measure the absolute abundance of the endogenous peptide of interest.

Liquid-Tissue-SRM has been developed which is a MS-based technology platform that measures the absolute abundance of targeted proteins in patient-derived FFPE tissue [30], [31]. In this method, laser microdissection is used to isolate tumor cells from FFPE sections and a cell lysate is prepared and digested into tryptic peptides (**Figure 1**). A known amount of a heavy peptide representing the targeted analyte is added to the lysate. Analysis using a SRM-MS method will then quantify the amount of analyte in the sample.

In this study we present a Liquid Tissue-SRM assay for measuring the absolute abundance (amol/µg) of Met expression in FFPE tumor tissues ungoing laser microdissection. We tested the reproducibility across sample replicates as well as between different instrument platforms and operators. We also tested Liquid-Tissue-SRM Met levels in serial tissue sections re-analyzed one year after the initial testing; lack of temporal reproducibility is a recognized limitation of IHC. We then applied the developed assay to measure Met levels in 130 GEC FFPE tumors (in both small endoscopic and core biopsies, as well as curative surgical resection specimens), and results were correlated with Met expression as either an H-score or as a binary (positive/negative) score as determined by IHC, as well as *MET* gene copy number (GCN) and *MET/CEP7* ratio as determined by fluorescence in situ hybridization (FISH). Finally, we sought to define a SRM expression level cut-off that would optimally distinguish between Met expression consistent with *MET* gene amplification as compared to expression without *MET* gene amplification.

## Results

### SRM assay development and preclinical validation

**Figure 2:**
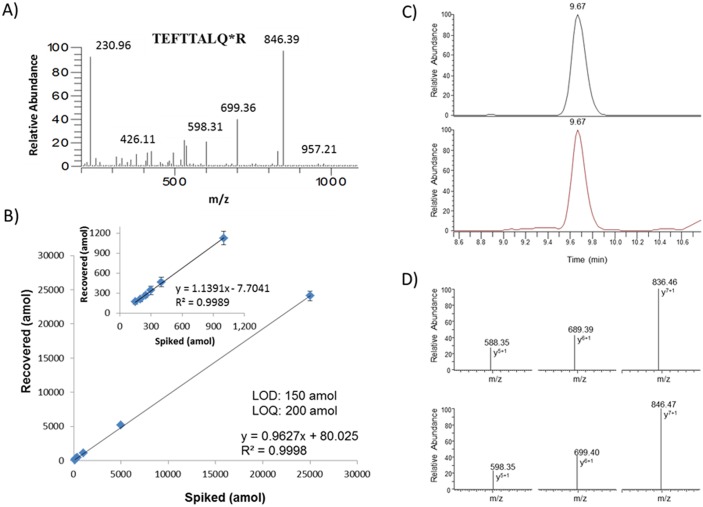
Development of SRM assay for Met showing the fragmentation spectrum for heavy TEFTTALQR peptide (A), the standard curve generated in human SK-BR-3 cell lysate (B); inset: the standard curve generated without the highest spiking point (25000 amol). The total ion chromatograms for the light and heavy isotopically labeled peptides are shown (C) along with the transition ions used to identify and quantitate each peptide (D).

For development of the Met Liquid Tissue-SRM assay, multiple peptides obtained from a tryptic digest of recombinant Met were measured using MS. The resulting three candidate peptides, NLNSVSVPR, GDLTIANLGTSEGR, and TEFTTALQR, were then extensively screened in multiple formalin-fixed cell lines and FFPE clinical samples. The peptide ^418^TEFTTALQR^426^ provided the most reproducible peak heights, retention times, chromatographic ion intensities, clean elution profile, and distinctive/reproducible transition ion ratios; therefore, this peptide was selected for clinical assay development. SRM transitions used for the quantification of Met was selected based on the representative MS fragmentation spectrum for peptide ^418^TEFTTALQR[^13^C_6_,^15^N_4_]^426^ (**Figure 2A**). Standard curves were generated to determine the assay’s limits of detection and quantitation (LOD and LOQ) by spiking various amounts of the light peptide into eight aliquots of lysate prepared from formalin-fixed (FF) SK-BR-3 cells containing consistent amount of the heavy isotopic version of the peptide. The final concentrations of light peptide in the lysates ranged from 150 to 25000 amol. The amount of light peptide added to each of the eight calibration samples along with the amount detected in each is listed in **Table 1**. Coefficients of variation (CV’s) for the various concentration points ranged from 2.8% to 20% for samples analyzed in triplicate. The amount of light peptide recovered (amol) was plotted against the amount of light peptide spiked (amol) to create a standard curve. The LOD and LOQ was 150 and 200 amol, respectively, with a linear regression value of R^2^ = 0.9998 (y = 0.9627x+80.025) for the assay standard curve when all 8 spiking concentration points were included (**Figure 2B**) and R^2^ = 0.9989 (y = 1.1391x–7.7041) when excluding the highest point of 25000 amol (**Figure 2B** **inset**). The standard curve showed linearity and low variations over the 2 orders of magnitude concentration range tested. The chromatographic profile of the light and heavy versions of the peptide TEFTTALQR are shown in **Figure 2C**. Both peptides eluted at 9.67 minutes within the SRM-MS analysis and the corresponding SRM transition ion ratios are shown in **Figure 2D**. The precursor ions for the light and heavy peptides are *m/z* 533.78 and 538.78, respectively and three fragment ions per precursor were chosen for the identification and quantification of Met. The fragment ions for the light and heavy peptides and their corresponding optimized collision energy are *m/z* 588.35 (19V)/689.39 (20V)/836.42 (19V) and 598.35 (19V)/699.39 (20V)/846.47 (19V), respectively.

**Table 1:** Characteristics of Met light peptide spiked SK-BR-3 lysates used to prepare calibration curve to determine limit of detection (LOD) and quantitation (LOQ) for development of Met Liquid Tissue-SRM assay.

Sample	Amount of MetPeptide Spiked(amol)	Amount of Met Peptide Recovered (amol)	SD (amol)	CV (%)
1	25000	24090.7	686.0	2.8
2	5000	5167.7	164.8	3.2
3	1000	1127.1	103.6	9.2
4	400	466.3	71.3	15.3
5	300	338.9	67.2	19.8
6	250	266.6	34.6	13.0
7	200	206.7	39.6	19.1
8	150	168.0	33.6	20.0

**Legend:** amol, attomol (10**^−^** ^18^ mol); SD, standard deviation; CV, coefficient of variation.

**Figure 3:**
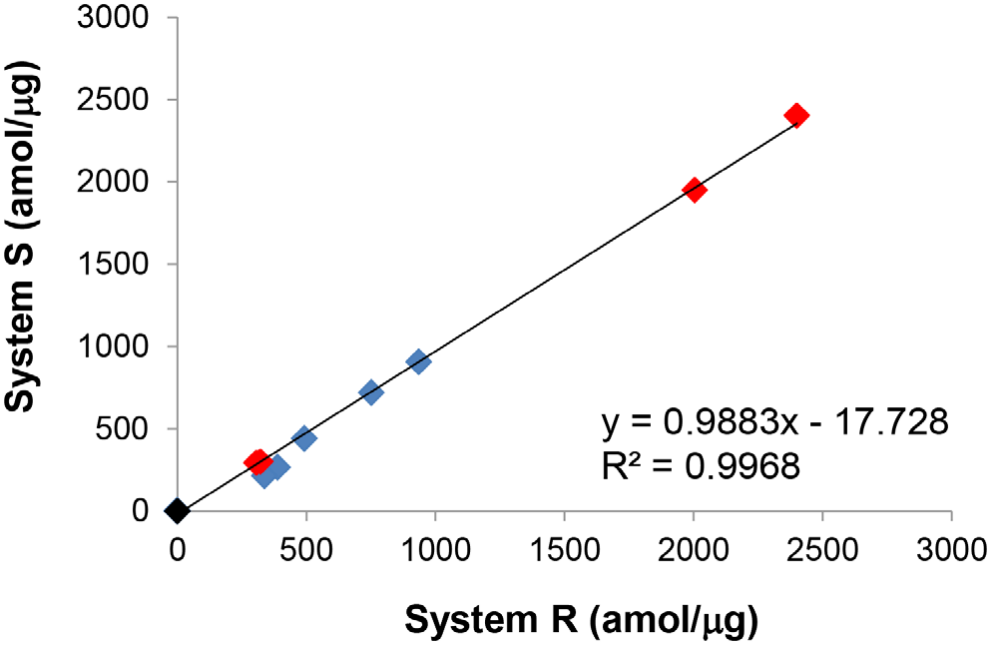
Precision assessment for measuring the absolute abundance of Met in 9 NSCLC and 11 GEC FFPE tissues. Each sample was analyzed on two different liquid chromatography-mass spectrometry systems operated by two different scientists. Red square: Met positive GEC tumors (4/11). Blue square: Met positive NSCLC tumors (5/9).

To test assay precision, Met was measured in 9 human NSCLC and 11 human GEC FFPE tissues. The precision study was conducted on two different LC-MS systems (System R and System S) operated by two different scientists. Five of the nine NSCLC samples and four out of eleven GEC tissues showed Met levels above the LOD. The levels of Met in these tissues ranged from 275.5–2401.2 amol/µg. The CVs for these measurements ranged from 1.5–17.7%. A plot (**Figure 3**) comparing the results from systems R and S had a linear regression value of 0.9968 (y = 0.9883x–17.728) demonstrating that the SRM-MS method generated extremely low level of variance between the two systems.

### Comparison of Liquid Tissue-SRM and ECL Met Quantitation

**Figure 4:**
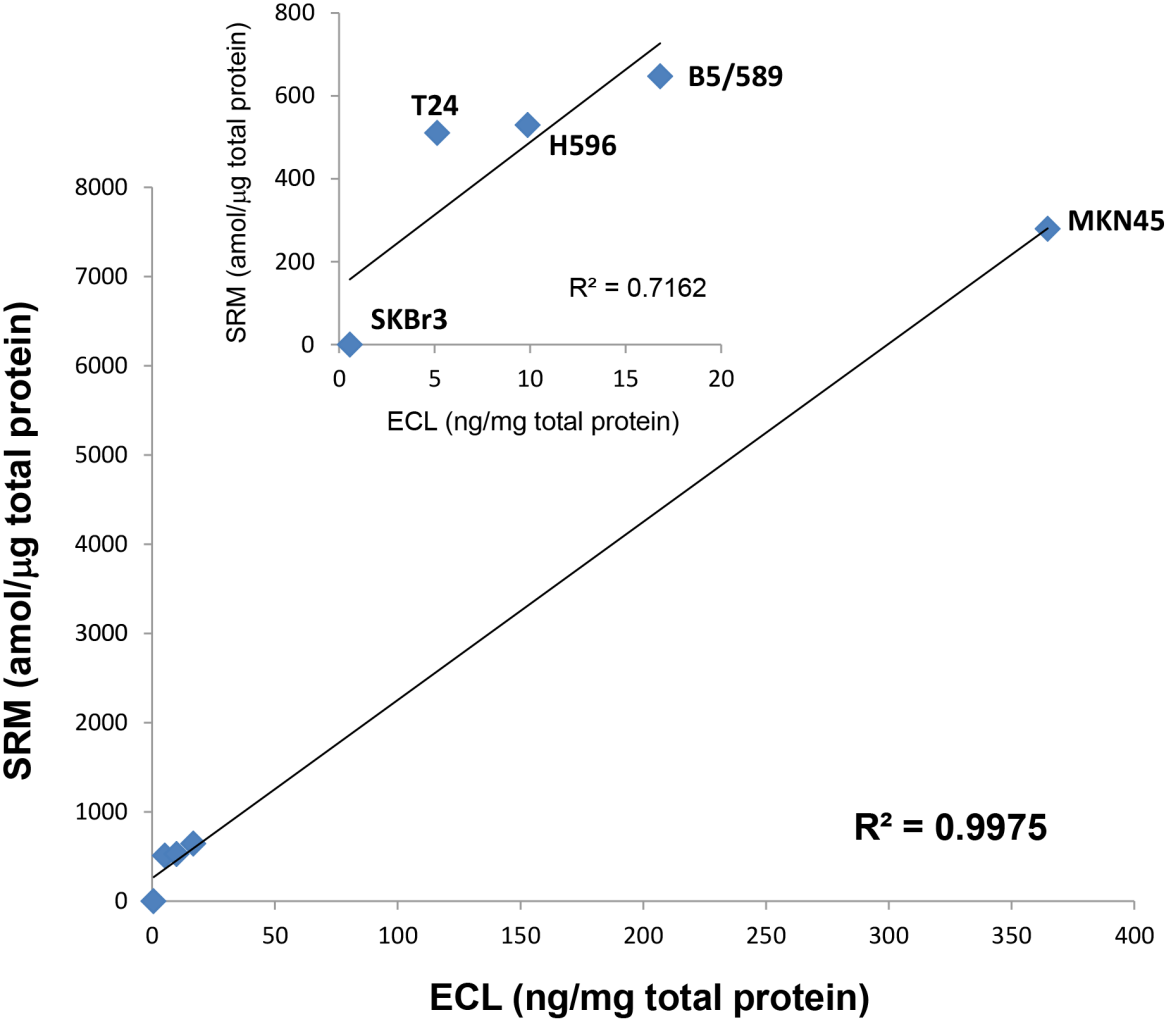
Comparison of Met levels measured in five different cell lines using Liquid Tissue-SRM and an electrochemiluminescent (ECL) immunoassay. Inset: comparison of SRM and ECL Met levels measured within the four cell lines containing the lowest concentration of Met.

The levels of Met in five different cell lines (B5/589, H596, SKBr3, MKN45, and T24) were measured using both Liquid Tissue-SRM and electrochemiluminescence (ECL) immunoassay. The results obtained using the two methods are plotted against each other in **Figure 4**. The correlation coefficient (R^2^) between the two sets of measurements was 0.998 when all five cell lines were compared, and 0.716 when excluding MKN45, a *MET* gene amplified line with extremely high expression. (**Figure 4** **inset**). Overall there was good correlation of the measurements provided by Liquid Tissue-SRM and ECL.

### Resistance of Liquid Tissue-SRM Met Assay to Preanalytical Handling and Time from Tissue Sectioning

**Figure 5:**
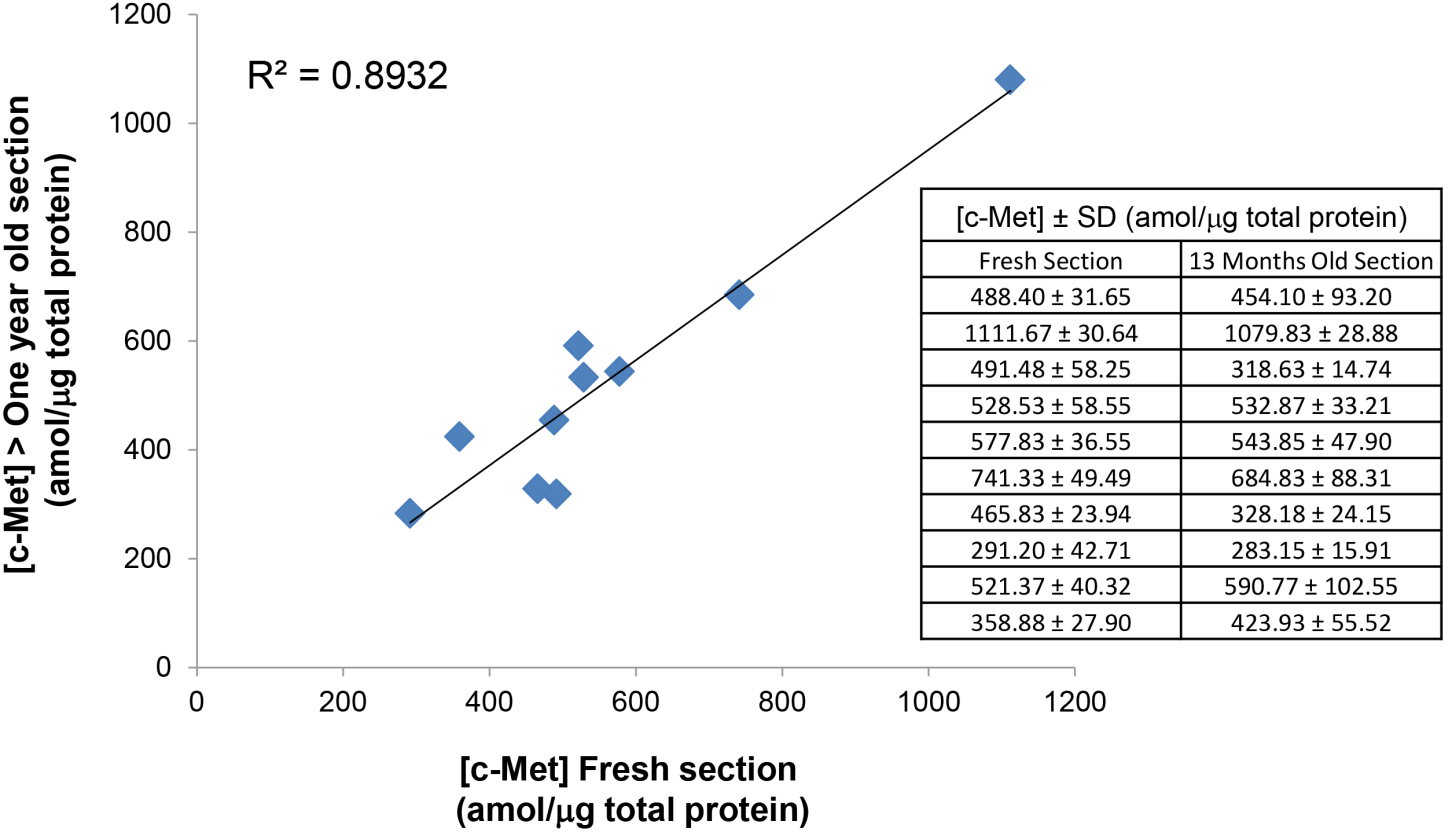
Temporal reproducibility of FFPE sections processed and analyzed using Liquid Tissue-SRM over one year apart. SD, standard deviation.

IHC is known to be very sensitive to preanalytical variables, including fixation time, warm and cold ischemia time, and the age of tissue sections. To test the robustness the SRM assay, Met protein expression was measured on tissues dissected from sections cut from ten NSCLC tumor blocks, where the first microdissection/analysis occurred immediately after sectioning and the subsequent microdissection/analysis from the adjacent serial sections occurred 13 months later. A plot comparing the Met levels detected in these paired tissues is shown in **Figure 5**. Met concentration values measured in these tissues at the different time points are also shown (**Figure 5** **inset table**). The correlation coefficient between these two groups of samples was 0.893, showing that the Liquid Tissue-SRM process provides reproducible results for FFPE samples that have been previously sectioned up to 13 months prior to analysis.

In a separate experiment, the levels of EGFR in A431 xenografts fixed for various lengths of time (4 hour-1 week) ranged from 2–3.01 fmol/ug; similarly, EGFR expressions ranged from 2.3–3.0 fmol/ug when compared between immediate and 1 hour-delay fixation. The CVs for these measurements ranged from 3.7–24%. This demonstrated the excellent assay robustness regardless of fixation time (4 hour–1 week), as well as prefixation cold ischemia (1 hour immediately before fixation).

### Met Expression in the Gastroesophageal Cancer (GEC) Patient Cohort

**Figure 6:**
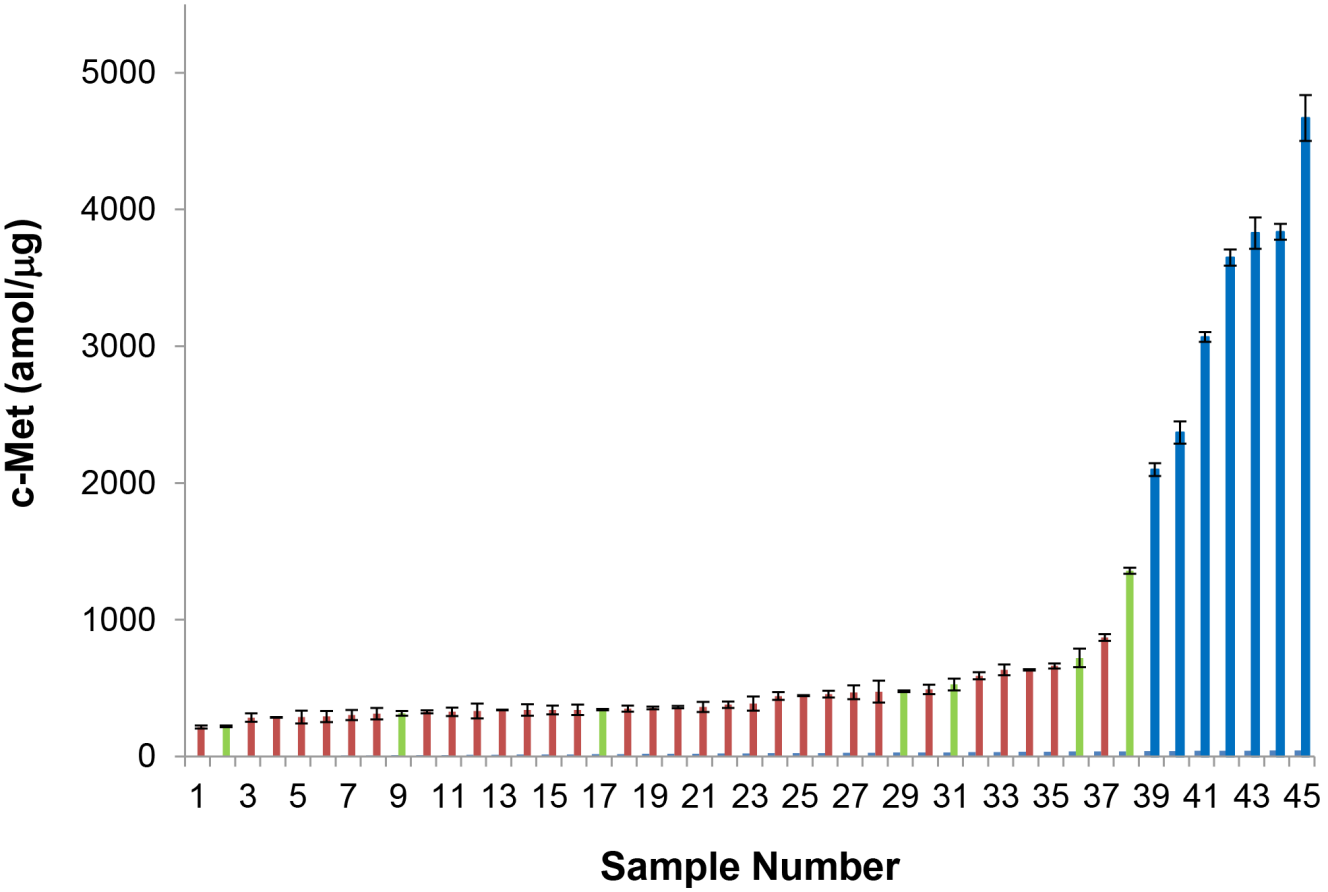
Absolute levels of Met in those GEC tumors above the LOD (45/130) as measured using Liquid Tissue-SRM. Blue indicates *MET* gene amplified samples (ratio of *MET/CEP7* ≥2) as determined by FISH, and green indicates those not gene amplified (ratio<2). Samples in red were not FISH tested. Sample 36 (526.93 amol/ug) is taken from reference 18 (sample obtained at disease recurrence after initial onartuzumab treatment).

Met levels were quantitated in a cohort of 130 GEC tumors, comprised of primary tumor resections, endoscopic primary tumor biopsies, and core needle biopsies of metastatic sites (liver, peritoneum) (**Table S1**). The Met levels in the tumors were above the LOD in 45/130 (34.6%) samples with the levels in these samples ranging from 215.88 to 4669.5 amol/µg (**Figure 6**). The presence of Met could be unequivocally detected above the LOD in four additional samples; however, as their quantities were below the LOQ they were labeled as “positive” ( = ∼150 amol/µg). While the majority of the samples had Met levels on the order of a few hundred amol/µg total protein, the concentration of Met in 7 tissues ranged from over 2000 amol/µg to almost 5000 amol/µg total protein. The CV for triplicate measurement of all samples was less than 20%. In 73% of the samples, the CVs were less than 10%.

### Comparison of Met SRM and MET gene copy number (GCN) in GEC cell lines & tissues

Met SRM results were compared with those of *MET* GCN in order to identify an optimal SRM cut-off level that was consistent with *MET* amplification (i.e. FISH ratio *≥*2 and GCN≥4, see methods). We first assessed 22 GEC cell lines and 1 lymphoblast control line for *MET* GCN by FISH, and Met expression by SRM (**Table S2, Figure S1**). Met levels were detected in 16/22 (72.7%) GEC lines, and ranged from 150 to 6290 amol/µg. FISH confirmed three previously reported *MET* amplified GEC lines (SNU5, MKN-45, and OE33) [4]. Using a cut-off value of 1500 amol/µg, derived from these data, SRM demonstrated a 100% (3/3) sensitivity (95% CI 0.29–1) and 100% (20/20) specificity (95% CI 0.83–1) in discerning *MET* amplification status. This preliminary cut-off was therefore used to evaluate 30 GEC tissue samples prospectively.

**Figure 7:**
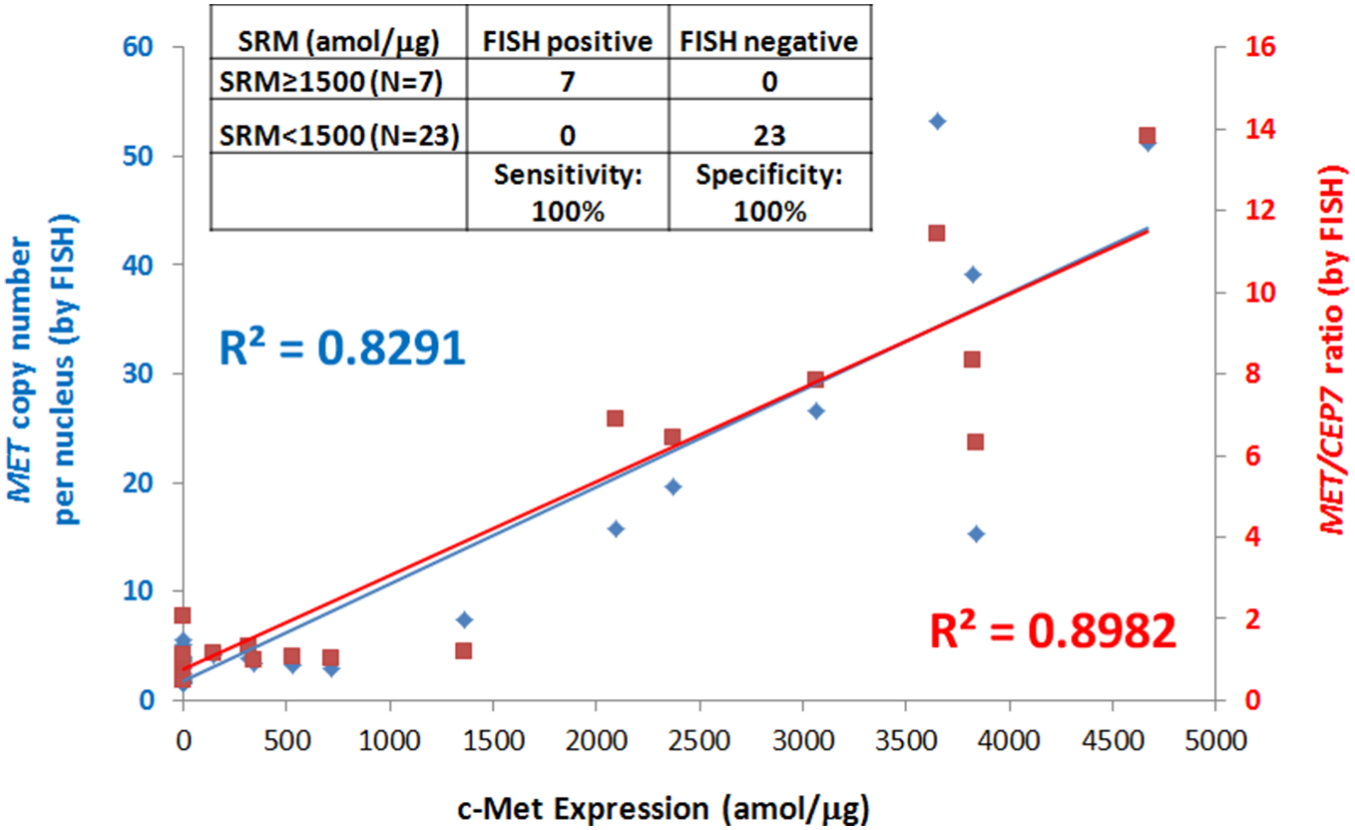
Correlation of Met levels using Liquid Tissue-SRM and *MET* gene amplification by FISH in 30 GEC tumors. This cohort includes diploid/low polyploid, high polyploid, and amplified samples. The left y-axis (blue diamond) represents the *MET* copy number per nucleus and the right y-axis (red square) indicates *MET:CEP7* ratio.

We performed *MET* FISH in a subset (n = 30) of the total 130 GEC cohort (**Table S3**). Twenty-three samples (23/30) had mean *MET* GCN per nucleus >2. Among these elevated GCN samples, 7 tumors (23%) had *MET* gene amplification (ratio *MET:CEP7* ≥2 and GCN≥4); the high rate of *MET* gene amplification was due to intentional enrichment in this 30 sample cohort with patients having previously established *MET* amplification by FISH. The Met SRM result for the same 30 cases is plotted against *MET:CEP7* ratio (red) or *MET* GCN (blue) (**Figure 7**). Met expression correlated well with both parameters in all but two samples (Sample 23 and 24). The correlation coefficient (R^2^) between the two sets of measurements in all 30 samples were 0.651 for SRM and *MET:CEP7* ratio, and 0.774 for SRM and mean *MET* GCN per nucleus (figure not shown). While Sample 24 had a *MET:CEP7* ratio of 1.18, it demonstrated high polysomy with a mean 7.35 copies of *MET* per nucleus, corresponding with higher expression compared to low polysomy/disomy samples, but lower than *MET* amplified samples. Sample 23 represented a primary gastric cancer; only ∼20–30% of the tumor cells revealed clustered gene amplification (*MET:CEP7* ratio 14.93) (sample 23a), while the remainder of tumor cells were low polysomic (sample 23b) (**Table S3, Figure S2**). Although the gene amplified subclonal area demonstrated a mean 41.8 copies of *MET* per nucleus, the remaining non-amplified tumor cells were also microdissected during processing for MS analysis based tumor cells observed by H&E staining. This tumor molecular heterogeneity, therefore, likely led to a lower ‘diluted’ SRM Met read. This explanation was verified by examining the metastatic lymph node of this patient (Sample 23c), where the hypothesis of possible clonal selection of the *MET* amplified cells from the primary tumor to the lymph node was evaluated. The metastatic lymph node (Sample 23c) did indeed reveal a mean of 15.33 *MET* copies per nucleus (ratio *MET:CEP7* 6.31) with 100% of the scored tumor cells having elevated *MET* GCN. The SRM detected a high amount of Met (3836 amol/µg) (**Table S3, Figure S2**).

After discounting this molecularly heterogeneous primary tumor sample 23a, and replacing with sample 23c, SRM and *MET:CEP7* ratio showed high correlation R^2^ = 0.898; SRM and *MET* GCN per nucleus had R^2^ = 0.829 (**Figure 7**). The SRM Met level of 1500 amol/µg, as determined from the cell line data, was 100% (7/7) sensitive (95% CI 0.59–1) and 100% (23/23) specific (95% CI 0.85–1) for discerning *MET* amplification status by FISH in the FFPE tissues.

Overall, the pooled data from the cell lines and GEC tissues (using 23c) demonstrated high concordance between *MET* GCN (or amplification ratio) and SRM Met measurement. SRM identified GEC lines and samples having *MET* amplification with 100% (10/10) sensitivity (95% CI 0.69–1) and 100% (43/43) specificity (95% CI 0.92–1).

### Comparison of Met IHC to SRM in GEC cell lines and FFPE tissues

**Figure 8:**
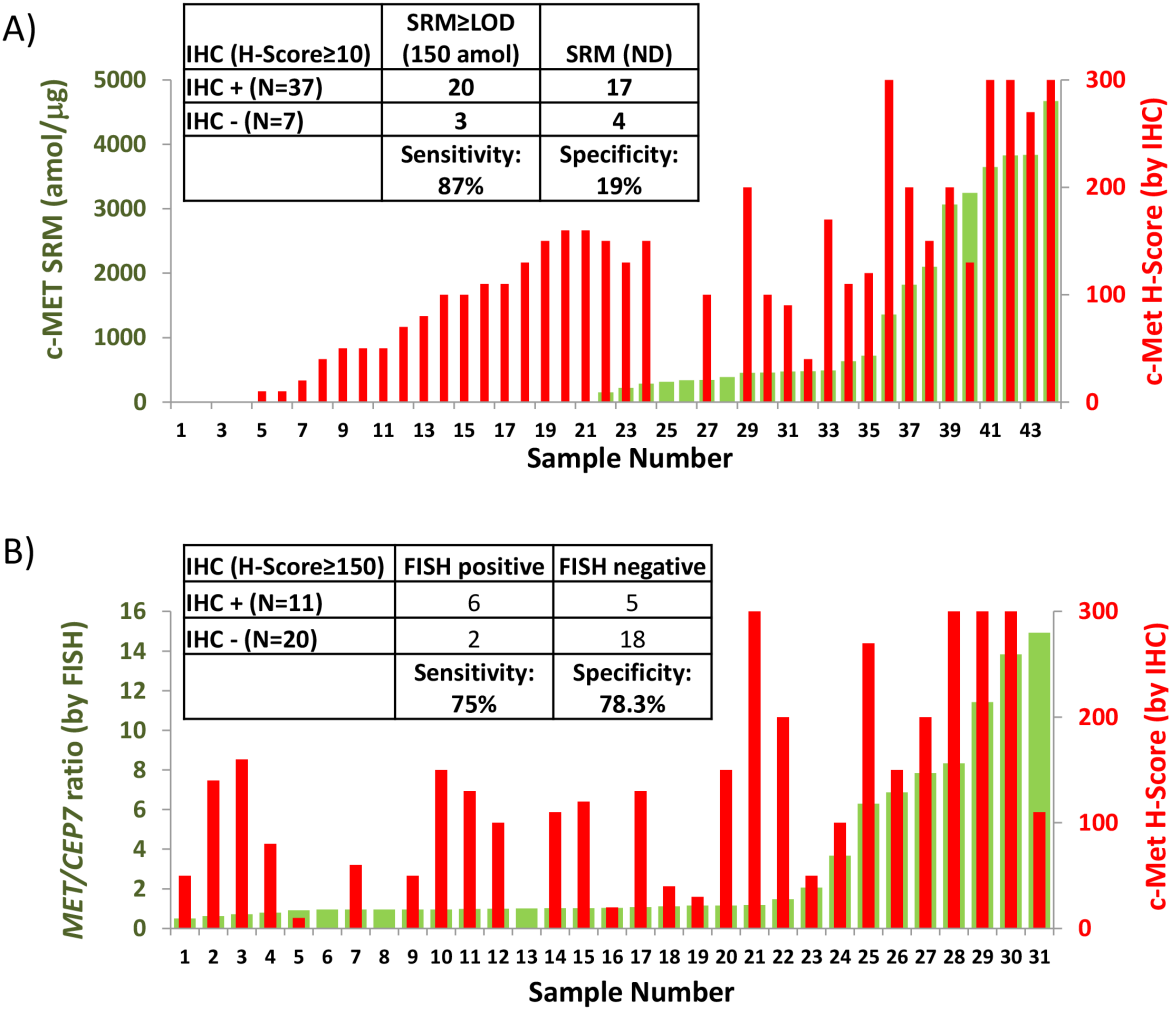
IHC to SRM or FISH a**ssay correlations.** Correlation of IHC Met positive H-score to (A) Met SRM (amol/µg) or (B) MET/CEP7 ratio by FISH. Inset tables assess sensitivity/specificity of IHC H-score, assuming SRM (A) and FISH (B) as the comparative standards.

We assessed Met IHC in various cell line FFPE pellets (**Figure S3**), which under-appreciated the expression intensity level differences between *MET* amplified and non-amplified lines. We then assessed the subset (n = 44) of the total 130 GEC cohort having both Met IHC and SRM. A comparison between the two in terms of *any* expression revealed a correlation coefficient R^2^ = 0.54 (**Figure S4**). Using an H-score of ≥10 and SRM level of ≥LOD (150 amol/ug) to define ‘Met+’, there was high discordance between the two assays (**Table S4**, **Figure 8A**). Using SRM as the reference, IHC identified 20/23 (sensitivity 87%; 95% CI 0.66–0.97) positive SRM samples. However, IHC was negative in only 4/21 SRM negative samples (specificity 19%, 95% CI 0.05–0.42). This was only marginally improved when using an alternate IHC scoring system where Met is considered positive when ≥25% of tumor cells stain with ≥1+ intensity (**Table 2**). Using this system, of the 23 positive SRM samples, IHC identified 19 positive (sensitivity 82.6%, 95% CI 0.61–0.95); of 21 negative SRM samples, IHC identified 7 negative (specificity 33.3%, 95% CI 0.15–0.57). Increasing the IHC cut-off to ≥50% tumor cells staining ≥1+ improved specifity to 42.9% (95% CI 0.22–0.66) at the expense of sensitivity 78.3% (95% CI 0.56–0.93), as would be expected. In summary, IHC and SRM for Met expression were relatively discordant.

**Table 2:** Comparison of Met IHC (≥25% or ≥50% positivity at any intensity ≥1+) with Met protein levels (by SRM) in 44 gastroesophageal cancer FFPE tissues.

Met IHC		Met SRM	
Scoring Criteria(% positive)	Status	≥150 amol (LOD)	Not Detected
IHC (≥25%)	IHC positive (N = 33)	19	14
	IHC negative (N = 11)	4	7
		Sensitivity: 82.6%	Specificity: 33.3%
IHC (≥50%)	IHC positive (N = 30)	18	12
	IHC negative (N = 14)	5	9
		Sensitivity: 78.3%	Specificity: 42.9%

**Legend:** IHC, immunohistochemistry; SRM, selected reaction monitoring; FFPE, formalin fixed paraffin embedded; LOD, limit of detection; amol, attomol (10**^−^** ^18^ mol).

### Comparison of Met IHC to MET FISH in GEC FFPE tissues

We assessed a subset of 31 GEC samples with both Met IHC and *MET* FISH scores to assess the sensistivy/specificity of H-score to identify *MET* amplified tumors (**Table S5**). Using an H-score of ≥150 and FISH ratio of ≥2 with GCN≥4 as ‘amplified’, there was discordance between the two assays (**Figure 8B**). The correlation coefficient comparing IHC to FISH was R^2^ = 0.48 (FISH ratio) and R^2^ = 0.51 (FISH GCN) (**Figure S5**). Using FISH as the gold standard, IHC identified 6 of 8 FISH amplified tumors resulting in a sensitivity of 75% (95% CI 0.35–0.97). IHC identified tumors with H-score ≥150 in 5 FISH negative samples resulting in specificity of 78.3% (95% CI 0.56–0.83). In summary, using an IHC H-Score of ≥150 to discern *MET* amplification status resulted in a sensitivity of 75% and specificity of 78%. Altering the H-score cut-off could increase the sensitivity at the expense of specificity, or vice versa. H-score was out-performed by SRM in the ability to accurately discern *MET* amplification status in this study.

### Comparison of Met IHC, SRM Met, and MET FISH in GEC tissues

**Figure 9:**
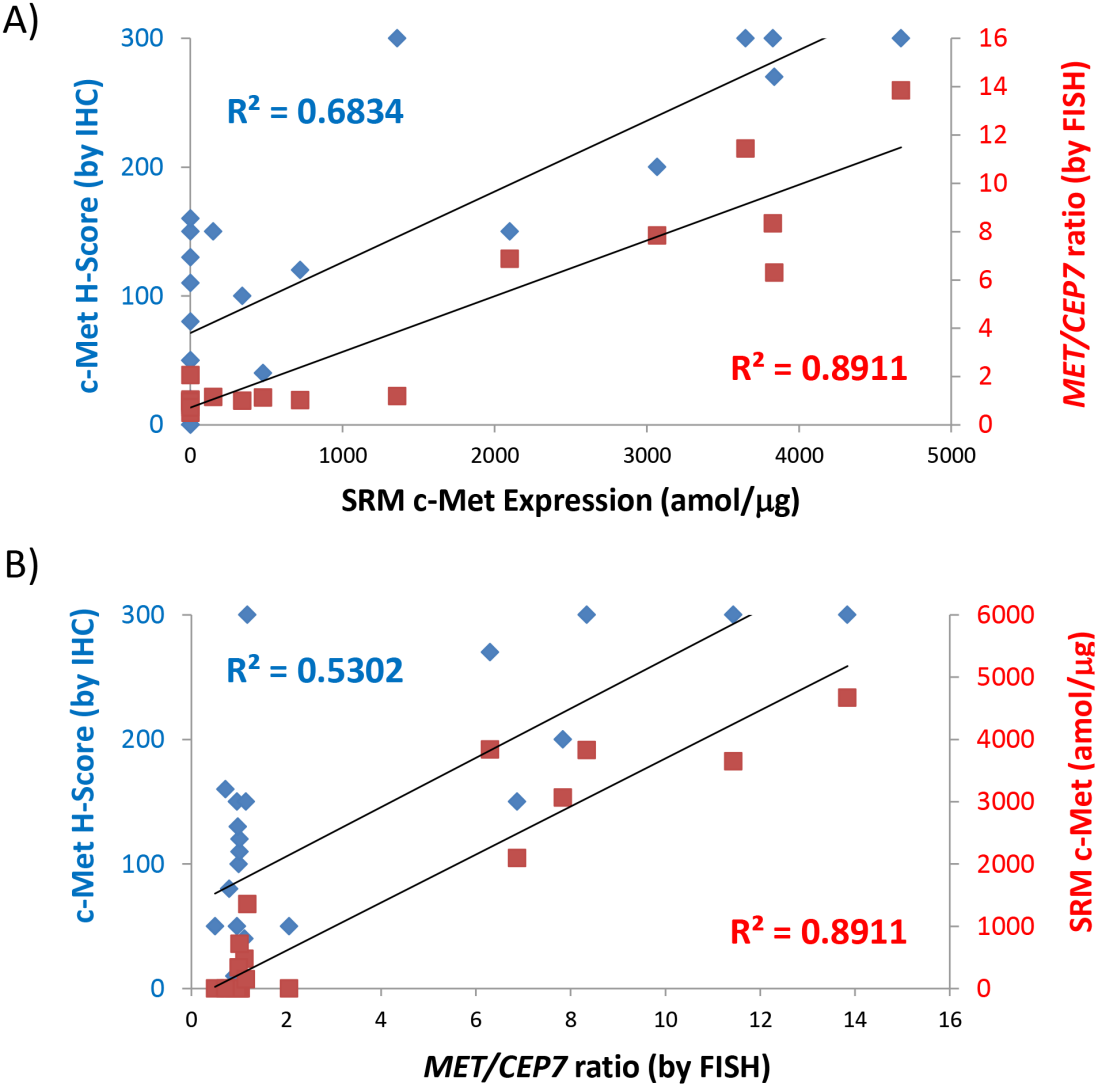
Three-way SRM-IHC-FISH assay correlations. (A) Correlation of SRM Met level (amol/µg, x-axis) to Met H-Score by IHC (blue, left y-axis) and to FISH *MET/CEP7* ratio (red, right y-axis) in GEC FFPE tissues having all three tests performed. Correlation coefficients for the comparisons are in their respective colors. (B) Correlation of FISH *MET/CEP7* ratio (x-axis) to Met H-Score by IHC (blue, left y-axis) and to SRM Met level (red, right y-axis) in GEC FFPE tissues having all three tests performed. Correlation coefficients for the comparisons are in their respective colors.

We assessed the correlation of IHC, SRM and FISH in 24 GEC FFPE tissues that had been tested using all three methods (**Table S6**). A stronger correlation of SRM with FISH *MET/CEP7* ratio (R^2^ = 0.89) was observed compared to SRM with IHC H-Score (R^2^ = 0.68) (**Figure 9A**). Similarly there was a stonger correlation of SRM with FISH *MET/CEP7* ratio (R^2^ = 0.89) compared to IHC H-Score with FISH (R^2^ = 0.53) (**Figure 9** **B**).

## Discussion

Detection of relevant protein biomarkers in tumor samples has been routinely accomplished through IHC analysis of FFPE tissues. While IHC has proven useful, it is limited by preanalytical variability, antibody specificity, imperfect sensitivity, low throughput, and inter-laboratory differences in methodology and analysis/scoring standardization.

IHC is used to semi-quantify proteins in clinical samples and can be a critically important feature that often dictates diagnostic or therapeutic decisions. The colorimetric signal produced by IHC, however, is nevertheless subjectively “quantitated” as interpreted by the Pathologist. To generate an IHC “score”, the staining intensity and percentage of cells must be taken into account as well as the extent of perceived background staining. Despite recent application of image software and signal quantitation hardware platforms for IHC analysis, the lack of standardization and subjective quantitation are challenges of IHC being uniformly, systematically, and successfully applied for characterizing target proteins in cancer patient tissues.

To overcome these limitations we have developed a targeted Liquid Tissue-SRM assay to measure Met directly from FFPE patient tissue. The assay requires only a small amount of sample, and is routinely accomplished using laser microdissection of a single 10 µm tissue section. Indeed, many of the GEC samples analyzed in this study were acquired as endoscopic or core biopsies of metastatic lesions. The assay is epitope-independent and therefore does not suffer from antibody-related issues such as degree of specificity, manufacturing variability, and tissue processing and fixation. Since the absolute quantity of Met in the sample is determined by software analysis of the MS signal, the result is objective and absolutely quantitative.

Archival FFPE sections are extremely valuable resources for translational clinical studies and for the validation of clinical utility for companion diagnostics. However, IHC analysis of previously cut tissue sections is limited, deeming its usefulness, reliability, and clinical significance on archival FFPE slides compromised over time. There was observed decrease in the reactivity for p53 in FFPE tissue sections that had been cut from a paraffin block and stored at room temperature for only two months [32]. Slides cut two months previously and stored at room temperature were stained simultaneously with those that had been freshly cut and results showed that for all cases, the immunoreactivity was weaker on slides that had been stored. In three instances where p53 staining was observed on freshly cut slides, no p53-immunreactivity was observed on the corresponding stored slides. This deterioration of immunoreactivity has been demonstrated to occur as early as 2 weeks after storage [33]. A loss of immunoreactivity has been reported for estrogen receptor (ER), progesterone receptor (PR), cyclin D1, E-cadherin, and Her2 on sections stored for 6 months at 4°C [34]. Most procedures for conducting IHC analysis recommend that sections be cut from the tissue block immediately before testing to preserve antigenicity. For example, Ventana recommends that sections stored longer than 6 weeks should not be used for IHC testing using its breast panel kit, which contains antibodies that target ER, Her2, PR, and Ki-67 (www.ventanamed.com).

Discordant result provided by older slides is a major concern, particularly due to the increasing importance of retrospective diagnostic tissue analysis. It is estimated that more than one billion FFPE tissue blocks are archived in tissue banks worldwide. Since the first description of formalin fixation and storage was described more than a century ago, this approach has become the technique of choice for preservation of tissue. Most importantly, in many cases previously cut sections are the only form in which remaining archived FFPE tissue is stored. Archiving the tissue should enable retrospective biomarker studies of various cancers; however, the temporal stability of IHC epitopes is unsatisfactory, with unreliable results. RNA expression analysis via next-generation sequencing (NGS) is also subject to wide variability, often lacking concordance with protein expression even in the best of circumstances, let alone when evaluating aged FFPE tissue sections [35], [36].

Simultaneous IHC analyses of multiple samples concurrently using tissue microarrays (TMAs) can be used to increase assay throughput, yet will often analyze tissues that have been stored for several weeks/months after sectioning from their paraffin block. Adjusting the IHC protocols for fresh or stored slides (e.g. different antigen retrieval methods) may offer a potential solution; however, this strategy eliminates the study’s consistency and introduces the presence of potentially undetectable artifacts that may arise by altering the protocol for a selected group of sections. Using different antibodies has also been suggested as a possible remedy to this problem. Different antibodies are known to provide highly variant results, even when used on identical slides [37]. Previous studies have also failed to demonstrate how storage conditions can be improved to alleviate the discrepancy between results provided by fresh and stored slides [33]. Finally, construction of TMAs in routine clinical settings is not performed for logistical reasons.

In our study we showed that Liquid Tissue-SRM results obtained at least one year apart of cut tissue sections are highly reproducible, providing a more robust platform for conducting both proteomic biomarker studies on archival samples. The robustness of Liquid Tissue-SRM results compared to IHC results is most likely due to the fact that the SRM analyte is a linear tryptic peptide instead of a three-dimensional epitope. As such, the SRM assay is less susceptible to preanalytical handling issues by heating the tissue to 95 degrees, followed by trypsinization. In addition to the age of cut sections, we have also assessed time of fixation (4 hour to 1 week) and cold ischemia (1 hour) prior to fixation. In each case, SRM results were similarly not impacted by these factors.

We then demonstrated a strong correlation with ECL expression and SRM for Met in FFPE cell lines. Next, analysis of 130 GEC samples using Liquid Tissue-SRM showed that Met at any level was detected in approximately 35% of the tumors. This rate of Met expression is similar to other studies, where Met ‘overexpression’ by IHC ranged from 23.7% to 70% [4], [38]–[40]. In addition to random patient selection, this wide variability of Met expression rates in the literature may be due to several factors, including a lack of standardization in the age of the tissue section, the staining procedure, and, very notably, the interpretation/scoring of the IHC analysis. Additionally, different antibodies (polyclonal and monoclonal) used for detecting Met also very likely contribute to the variability in Met expression rates observed across these different studies. On the other hand, studies in our MS lab have shown high intra-laboratory reproducibility across different operators and MS instrumentation (**Figure 3**), and this level of reproducibility should easily translate to other laboratories since the Liquid Tissue-SRM process does not rely on affinity reagents or subjective interpretation of the MS results. Finally, as demonstrated by Sample 23 in our series, intratumoral molecular heterogeneity (through space within the primary tumor, or from primary tumor to lymph node and/or distant site) may also contribute to variable rates of Met overexpression (as well as variable gene copy number). Therefore the site of the tumor sample should also be taken into account when determining the rate of Met overexpression within a given patient cohort, since metastastatic lesions may possess higher Met expression levels [41].

Gene copy number (GCN) elevation, and more specifically *MET* gene amplification have been shown to be both prognostic and predictive in preclinical and early phase clinical trials [4], [5], [11]–[14], [18]. FISH assessment of GCN is low-throughput, laborious, and subjective, similar to IHC. It is possible that certain Met inhibitors may be more efficacious in *MET* amplified tumors (eg. TKIs) while other inhibitors may be more efficacious in *MET* non-amplified tumors (eg. HGF ligand antibodies). Therefore, a multiplex analysis using SRM, including Met and other relevant proteins, that is able to provide a linear expression level of Met, as well as validated cut-off levels consistent with gene amplification, would be an ideal ‘one-time’ clinical test for economical use of scarce tissue. Moreover, the level of gene amplification and consequent level of overexpression may also be important with respect to prognostic and predictive benefit, as recently was shown for *HER2*/Her2 [42]. We demonstrated a strong linear correlation with Met SRM levels and both *MET* GCN and *MET/CEP7* ratio in cell lines and FFPE tissues, while IHC poorly correlated with SRM and FISH analyses. We demonstrated that ≥1500 amol/µg of Met had excellent (100%) sensitivity and specificity in all samples evaluated to detect *MET* FISH amplification, and further validation of this cut-off is ongoing prospectively in an independent large patient tumor cohort. One case (23a) was initially determined to be negative for Met expression by SRM and IHC, as well as non-amplified by FISH, NGS, and array comparative genomic hybridization (aCGH) (data not shown). However, a lymph node metastasis of this patient was shown to be highly amplified by both SRM and FISH, suggesting that the primary tumor harbored a subclonal population of *MET* amplified cells which seeded the lymph node metastasis. This intra-tumoral molecular heterogeneity underscores the need to molecularly profile lesions (metastases) that will ultimately become the most detrimental to the patient. This occurred in only 1 of 7 (15%) identified *MET* amplified FFPE samples; the true rate at which this observed tumor evolution occurs with respect to *MET* amplification is an area of active research in our laboratory. Efforts to biopsy metastatic lesions for molecular profiling may help limit biomarker misclassification due to intra-patient tumor molecular heterogeneity through space [41]. ‘High’ expression in non-amplified samples, albeit lower than *MET* amplified tissues, was observed with high polysomy samples (eg sample 24), representing a subset of samples having expression levels less than 1500 amol/µg but more ∼1000 amol/µg. Thus, ‘gene dosage’ clearly corresponded with SRM-MS Met expression levels in the cell lines and FFPE samples evaluated. Prospective evaluation of the prognostic and predictive properties of each of the observed expression groups (none: <150 amol/µg, low: 150–1000 amol/µg, high: 1000–1500 amol/µg, very high: >1500 amol/µg) will be valuable.

One limitation of this study is the use of a polyclonal anti-MET antibody for IHC testing. An ongoing study evaluating currently available monoclonal anti-MET antibody companion diagnostics will evaluate the performance of those antibodies compared to SRM and FISH assays. Another limitation is the current lack of correlation of the three assays (FISH, IHC, and SRM) with clinical outcome data (prognostic or predictive); this analysis is also ongoing. Despite a promising randomized phase II trial [43], a recent press release regarding anti-Met therapy in the phase III trial for ‘MET+’ lung cancer showed futility, and may hinge on the manner in which ‘MET+’ expression is defined; the ability of SRM to provide absolute expression that is linear and quantitative may overcome challenges posed by the subjectivity of IHC scoring [44]. Ultimately, after direct comparison, the companion diagnostic that is able to reliably quantify Met/*MET* with corresponding clinical relevance (prognostic and/or predictive) in a timely, tissue-economic, and cost-efficient manner will prevail.

As biomarker discoveries and the establishment of their clinical relevance continue, it will be critical that the companion diagnostic assays be accurate but also expandable. While assays used to determine biomarker expression will be performed using many different technologies (e.g., MS, IHC, FISH, DNA/RNA-seq etc.), this expansion must not only preferably include the ability to multi-plex additional important analytes within the assay [45], [46], but should also be able to translate easily to other tumor types. Since the Liquid Tissue-SRM method developed for Met requires no antibodies or other types of affinity reagents, and is performed using an unbiased sample preparation method, this ‘next-generation’ proteomic companion diagnostic is easily applied to other tumor types besides GEC. Moreover, additional SRM processes able to simultaneously quantitate up to 80 protein biomarkers from limited tissue samples have been developed, along with Met, in a single assay from a single 10 µm tissue section cut onto a microdissection slide [30], [31], [41], [45]. The ability to provide the protein expression data of many actionable biomarkers will enable better economic use of these scarce clinical tissue samples as well as more accurate multivariate prognostic/predictive capabilities [47], [48]. Upon receiving a tissue sample, generation of a quantititative report is on the order of 5 days, which competes with IHC turnaround time - particularly when taking into account the numerous advantages of SRM compared to IHC discussed above. We are currently evaluating the companion diagnostic SRM ‘GEC-plex’ [45], including Met, in a large clinically linked series of GEC samples as a prognostic and predictive tool, and to further evaluate the cut-off level of ≥1500 amol/µg for *MET* amplification as a surrogate for ‘Met-addicted’ tumors. Additionally, the ‘GEC-plex’ is incorporated as an integral diagnostic test, along with targeted NGS, to prospectively assign GEC patients into predefined molecular subsets in the planned PANGEA clinical trial [41].

## Materials and Methods

### Ethics Statement Human Samples

All research involving human tissue samples was approved by the University of Chicago Review Board (IRB16146B and IRB16294A) and conducted in accordance with these protocols and the Declaration of Helsinki. Written informed consent from the donor or next of kin was obtained in accordance with the tissue procurement protocols.

### Tissue and laser microdissection

Tumor tissue was obtained from patients with gastroesophageal cancer (GEC) from the University of Chicago (Chicago, IL) dating between 1999 and 2013 (N = 130). Of the samples evaluated, 63% were from curative intent resection specimens of the primary tumor, 23% were from endoscopic biopsy of the primary tumor via upper endoscopy, 7% were from intraoperative biopsy of peritoneal carcinomatosis, and 7% from ultrasound-guided core needle biopsy of metastatic lesions (liver 5%, peritoneum 2%). Surgically resected NSCLC tumor tissue (N = 19) was provided by Dr. Ignacio Wistuba and Dr. Jaime Rodriguez-Canales (MD Anderson Cancer Center). For measurement of SRM Met abundance, tissue sections or FFPE cultured cell sections (10 µm) were cut from blocks onto Director slides and deparaffinized for microdissection as described previously [31]. A cumulative area of a 12 mm^2^ containing approximately 45,000 malignant cells was microdissected by laser microdissection from each FFPE cancer specimen and cultured cell lines.

### Cell culture and reagents

The human GEC lines were obtained and cultured as previously described [4]. These included CP-A, CP-B, CP-C, CP-D, SNU-1, AGS, CAT-2, HGC-27, MKN-1, NCI-N87, OE19, OE33, SNU-5, MKN-45, Hs746t, KATOIII, and SNU-16. CAT lines were established from ascites aspirates from patients at the University of Chicago under preapproved guidelines and IRB protocols. For ECL assay: SK-BR-3 was obtained from ATCC and cultured as previously described [30]; the other four cell lines: B5/589, H596, MKN45, and T24 were maintained in RPMI 1640 medium. Procedures to prepare cell materials for SRM and ECL analysis were previously described [30].

### Cell Pellet Clots

Cells were collected and prepared in clots according to the procedure developed by Dr. Elizabeth Hyjek in the Department of Pathology at the Unversity of Chicago (unpublished) for formalin fixation and paraffin embedding for use in immunohistochemical (IHC) studies. After washing cells in Ca*^2+^*/Mg^2+^ deficient PBS, cells were collected and pelleted. Cells were resuspended in fibrinogen from human plasma (Sigma, St. Louis, MO) in complete media and clotted using thrombin (bovine origin) (King Pharmaceuticals, Bristol, TN). After overnight formalin fixation, the cell clots were paraffin embedded and mounted on slides in 4µm sections for IHC, as described below.

### SRM Assay Development

For development of the Liquid Tissue-SRM assay, recombinant Met protein (UniProtKB accession number P08581) was digested with trypsin and the resultant peptides analyzed using a TSQ Vantage triple quadrupole mass spectrometer (Thermo Scientific, San Jose, CA) equipped with a nanoAcquityLC system (Waters, Milford, MA). Software programs Pinpoint 1.0 and Xcalibur 2.1 (Thermo Scientific, San Jose, CA) were used to identify the optimal tryptic peptides for SRM analysis based on reproducible peak heights, retention times, chromatographic ion intensities, and distinctive/reproducible transition ion ratios. Peptides containing methionine or cysteine-residues were excluded due to their propensity to undergo unpredictable oxidation. The peptide TEFTTALQR, comprising residues 418–426 within the protein’s extracellular domain, was found to be unique to Met by comparing this sequence to the entire human proteome using the BLASTP function within the BLAST search engine (http://blast.ncbi.nlm.nih.gov/Blast.cgi), and sequence analysis using Phosphosite.org. Light (TEFTTALQR) and heavy (TEFTTALQR[^13^C_6_,^15^N_4_]) versions of this peptide were synthesized to develop and perform the assay (Thermo Scientific, San Jose, CA). SRM transitions used for the quantification of the light Met peptide were 533.78/588.35 (y^5^), 689.39 (y^6^), and 836.42 (y^7^) (Q1/Q3) and the transitions used for the heavy internal standard were 538.78/598.35 (y^5^), 699.39 (y^6^), and 846.47 (y^7^) (Q1/Q3).

The following mass spectrometer conditions were used for the SRM assays: Q1(FWHM); 0.4, Q3(FWHM):0.7, dwell time; 10 ms. Peptides were loaded onto a 75 µm inner diameter (i.d.)×1 cm IntegraFrit (New Objective, Inc., Woburn, MA) column and separated analytically using a 100 µm i.d.×12 cm PicoFrit (New Objective, Inc.) column slurry packed in-house with Jupiter Proteo reversed phase particles (C12, 4 µm, 90 Å pore size; Phenomenex, Inc., Torrance, CA). Peptides were eluted into the mass spectrometer using the following gradient: load onto pre-column for 3 min with buffer A (0.1% formic acid) at a flow rate of 5 ul/min and eluted with buffer B (0.1% formic acid, 99.9% acetonitrile) using a step gradient at 800 nl/min. Buffer B was increased from 1–9% (2 min), 9–15% (6 min), 15–25% (4 min), 25–50% (2 min), and 50–95% (1 min). Finally, the column was cleaned with buffer B for 3 min and equilibrated with buffer A for 13.5 min.

### Generation of Standard Curve

A standard curve was developed by serial dilution of the light peptide against a constant concentration of heavy peptide (5000 amol) added to the proteome extracted from human breast epithelial SK-BR-3 cells (ATCC HTB-30). Light peptide was added to the eight distinct lysates prepared from formalin-fixed SK-BR-3 cells to provide a final concentration ranging from 150 to 25000 amol (10^−18^ mol) (**Table 1**). The amount of light peptide recovered was plotted against the amount of light peptide spiked in to create a standard curve (**Figure 2**). All three product ions were used in the quantitation of endogenous Met. The data for the standard curve was acquired on a TSQ Vantage system utilizing the following conditions; Q1(FWHM):0.4; Q3(FWHM):0.7; dwell time: 10 ms. Each sample was analyzed five times. To determine the limit of detection (LOD) and limit of quantitation (LOQ), data was analyzed using Pinpoint 1.1. The LOD was determined by identifying the lowest concentration in the standard curve where the transition ion ratios and co-elution profile of the light synthetic peptide were similar to heavy synthetic peptide. Additionally, a signal to noise ratio >3 and a CV from quintuplicate measurements ≤20% were used. The LOQ was determined by identifying the next highest concentration of the standard curve above the LOD with a CV≤20% and signal to noise ratio >10.

### Statistical Analysis

The absolute abundance of SRM Met in each sample was calculated by measuring the area under the curve (AUC) for the endogenous and heavy standard peptide. The concentration of endogenous Met peptide was calculated using the following formula:
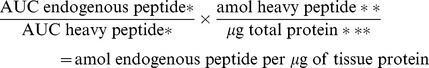

*Summation of peak area for 3 transitions from endogenous or heavy peptide.
**Quantity of spiked heavy internal standard (amol) injected.
***Quantity of total protein injected.

Light and heavy AUCs were exported from Pinpoint 1.1. Correlation linear regression coefficients were estimated using Excel. Patient samples were analyzed in triplicate, and results were charted as mean ± standard deviation (represented by error bars).

Confidence intervals of sensitivity and specificity for identifying *MET* amplification were determined using STATA 12.1 software.

### Evaluation of Assay Precision

To demonstrate the precision of the assay, nine human NSCLC and eleven human GEC FFPE tissues were analyzed independently by two different operators on each of two platforms (“System R” and “System S”) on different days. “System R” was comprised of a nanoAcquity LC coupled to a TSQ Vantage mass spectrometer and “System S” was comprised of a Thermo Easy nLC II coupled to a separate TSQ Vantage mass spectrometer. Each sample was run in triplicate on both systems.

### Comparison of Liquid Tissue-SRM and ECL

The concentrations of Met in five cell lines (B5/589, H596, SKBr3, MKN45, and T24) were measured using both Liquid-Tissue-SRM and an electrochemiluminescence (ECL) immunoassay. For the Liquid-Tissue-SRM study, the cells were cultured and formalin-fixed (FF) for 5 minutes. Lysates were extracted and tryptically digested from the FF cells using the Liquid-Tissue protocol and reagents. After addition of the Met internal standard, the samples were analyzed using SRM as described above. For the ECL immunoassay measurements, lysates were prepared from unfixed cells. ECL immunoassay of Met content was performed as described in [49]. Briefly, streptavidin coated 96-well plates (Meso Scale Discovery, Gaithersburg, MD) were coated with I-Block (Applied Biosystems/Life Technologies, Grand Island, NY), washed with PBS, coated with anti-Met antibody (R&D Systems, Minneapolis, MN), and washed again with PBS before adding samples or standards. Samples were prepared by extracting cultured cells in cold buffer containing non-ionic detergents and protease and phosphatase inhibitors; extracts were clarified by high speed centrifugation. Plates were washed with PBS before adding detection antibody (R&D Systems) and then again before adding Read Buffer and reading in a SectorImager 2400 (Meso Scale Discovery). The Met levels in each cell line were measured in triplicate for both Liquid Tissue-SRM and ECL studies.

### Assessment of Temporal Reproducibility of Met SRM-MS Assay

Tissue sections were cut from 10 NSCLC tumor blocks and microdissection via laser microdissection performed to collect cancer cells. Liquid Tissue lysates were immediately prepared from dissected cells from all three sections according to the manufacturer’s recommendations (OncoPlexDx, Rockville, MD – formerly Expression Pathology, Inc.). Total protein content for each Liquid Tissue lysate was measured using a Micro BCA assay (Thermo Fisher Scientific Inc, Rockford, IL). For SRM-MS analysis, 1 µg of the total protein extracted via laser microdissection from the tumor was injected onto the column. The MS and chromatography conditions were identical to those described above. Each sample was analyzed in triplicate. Approximately 13 months later, dissection was performed on the serial tissue sections and Liquid-Tissue lysates were prepared from each microdissected cell population. Met levels were measured by SRM-MS as described above.

### Measurement of Met in Clinical Samples

Liquid-Tissue lysates were prepared from GEC tumors microdissected from 130 FFPE tissues according to the manufacturer’s recommendations (OncoPlexDx, Rockville, MD). Total protein content for each Liquid-Tissue lysate was measured using a Micro BCA assay (Thermo Fisher Scientific Inc, Rockford, IL), blinded to IHC and FISH results. After addition of 5 fmol of heavy isotopically labeled internal standard to the sample, 1 µg of the total protein was injected onto the column. The MS and chromatography conditions were as described above. Each GEC Liquid-Tissue lysate was analyzed in triplicate.

### Fluorescence in situ hybridization (FISH)

Dual-color FISH assay was conducted, scored and interpreted on GEC cell lines and FFPE GEC tissues as previously described [4], [18], blinded to SRM and IHC results, using the following probes: *MET/CEP7* probe mixture containing homebrewed *MET* DNA (BAC clone RP11-163C9; 7q31.2) and alpha-satellite DNA clone pZ7.5 (centromere enumeration probe for chromosome 7) labeled with SpectrumOrange and the SpectrumGreen dUTPs, respectively, using the Nick Translation Kit (Abbott Molecular, Des Plaines, IL) [50]. The mean *MET* gene copy number (GCN) per nucleus, the mean *CEP7* GCN per nucleus and the *MET/CEP7* ratio were reported. FISH *MET* gene amplification in this study was defined as both *MET/CEP7* ratio ≥2 *and* GCN≥4. Both criteria were required to be met to rule out samples with *MET/CEP7* ratio ≥2 merely due to isolated loss of *CEP7*. These criteria were intended to be more stringent than using only the ratio, and were based on previous reports for MET GCN assessment and clinical correlation [5], as well as the authors’ observations that these samples do not have corresponding overexpression of Met (assayed with IHC or MS).

Alpha-satellite DNA clone pZ7.5 (*CEP7,* centromere enumeration probe for chromosome 7) (Archidiacono N, Antonacci R, Marzella R, Finelli P, Lonoce A, Rocchi M: Comparative mapping of human alphoid sequences in great apes, using fluorescence in situ hybridization. Genomics 25: 477–484 (1995)) labeled with SpectrumOrange and the SpectrumGreen dUTPs respectively using the Nick Translation Kit (Abbott Molecular, Des Plaines, IL).

### Immunohistochemistry (IHC)

IHC was described in our previous work [4], [18]. Briefly, IHC staining was performed using HRP-labeled dextrose-based polymer complex bound to secondary antibody (DAKO Cytomation, Carpinteria, CA). Then, 4 µm tissue sections were incubated for 1 h at room temperature with the rabbit polyclonal antibodies against Met (Zymed, 1∶100). Scoring was performed by an experienced pathologist based on intensity (0 none, 1 low, 2 intermediate, 3 high), blinded to the clinical data and FISH/SRM results, determined based on previous studies and the pathologist’s expertise. Descriptive patterns such as extensity of tumor (e.g., Diffuse versus patchy/focal and % or cells staining at each intensity), cellular localization of staining (membranous, cytoplasmic and nuclear) and tissue localization (invasive front versus central) were documented.

Additionally, an H-score was reported as an aggregate of the percent of tumor cells staining at each intensity for a score between 0 and 3 as follows:
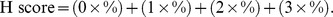
Binary H-Score to determine ‘MET positive’ was defined as an H-Score ≥10 and negative was H-Score <10.

Binary scoring (positive vs. negative) was also performed at higher cut-offs, where positive (MET+) was defined as any staining intensity ≥1+ in i) ≥25% of tumor cells (essentiall, an H-score ≥25), or ii) ≥50% of tumor cells (essentially, an H-score ≥50), as each of these is currently being assessed in ongoing clinical trials as potential IHC scoring systems of predictive benefit to anti-Met therapy.

## Supporting Information

Figure S1 Quantification of Met SRM-MS (amol/ug protein of sample loaded) for 22 GEC lines and a lymphoblast control line (GM15677*). *MET* amplified cell lines are red bars and double starred (**).
(PDF)

Figure S2 Correlation of IHC Met levels in the heterogeneous tissue sample #23 with *MET* FISH. The primary tumor (top row) was comprised of 75% IHC Met negative cells (GEC23b) and 25% Met positive (GEC23a), which correlated with FISH gene copy number (insets). The metastatic lymph node (bottom row, 23c) showed Met expression in 100% of cells, and tumor cells were all amplified by *MET/CEP7* ratio >2 (Met IHC low and high power). (see Table S3 for FISH scores and Met SRM values.).
(PDF)

Figure S3 Met IHC (bottom rows) for gastroesophageal cancer (GEC) cell line paraffin embedded pellets. For *MET* amplified lines (OE-33, SNU-5, and MKN-45) the difference of Met expression with non-amplified lines is under-appreciated by IHC, in contrast to levels observed with Mass Spectrometry (SRM-MS) (Table S2, Figure S1). RON tyrosine kinase, the other member in the MET tyrosine kinase family, is also demontrated with N-terminal, C-terminal, and phospho-RON (p-RON) antibody expression. Genomic characteristics of cell lines are represented above each cell line; GEJ, gastroesophageal junction.
(PDF)

Figure S4 Correlation of Met levels using Liquid Tissue-SRM and Met H-score by IHC in 44 GEC tumors. Inset: comparison of SRM and Met H-score in 8 GEC tumors where Met expression level ≥1500 amol/mg.
(PDF)

Figure S5 Correlation of Met H-score and *MET* gene amplification by FISH in 31 GEC tumors. The left y-axis (blue diamond) represents the *MET* copy number per nucleus and the right y-axis (red square) indicates *MET:CEP7* ratio.
(TIF)

Table S1 Levels of SRM Met observed in 130 gastroesophageal cancer FFPE tissues.
(DOCX)

Table S2 Met protein level detected by SRM and *MET* GNC detected by FISH in GEC cell lines.
(DOCX)

Table S3 Met protein level detected by SRM and *MET* GNC detected by FISH in 30 GEC FFPE tissues.
(DOCX)

Table S4 Met expression by IHC and SRM in 44 GEC FFPE tissues.
(DOCX)

Table S5 Met expression by IHC and *MET* GCN by FISH in 31 GEC tissues.
(DOCX)

Table S6 Met expression by IHC, SRM and *MET* GCN by FISH in 24 GEC tissues.
(DOCX)
