# Supplementary figures and images for: Absolute Quantitation of Met Using Mass Spectrometry for Clinical Application: Assay Precision, Stability, and Correlation with MET Gene Amplification in FFPE Tumor Tissue
Source: PLoS One. 2014 Jul 1;9(7):e100586. doi: 10.1371/journal.pone.0100586 (PMC4077664; doi:10.1371/journal.pone.0100586)

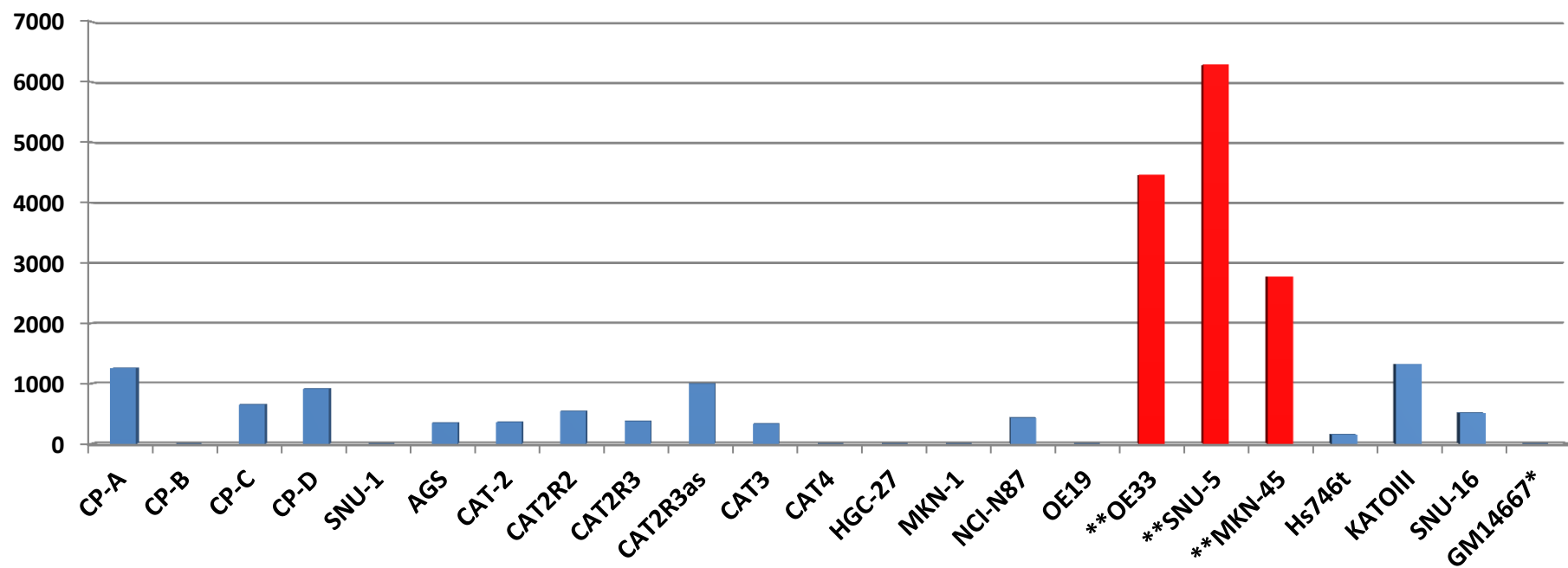

Supplement: Figure S1 — Quantification of Met SRM-MS (amol/ug protein of sample loaded) for 22 GEC lines and a lymphoblast control line (GM15677*). MET amplified cell lines are red bars and double starred (**). (PDF) [file pone.0100586.s001.pdf]

**IHC**

**FISH**

**Primary  
Tumor**

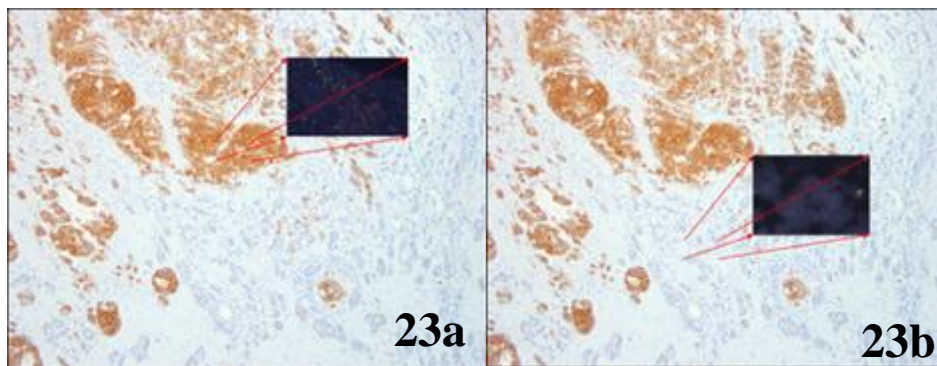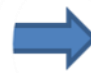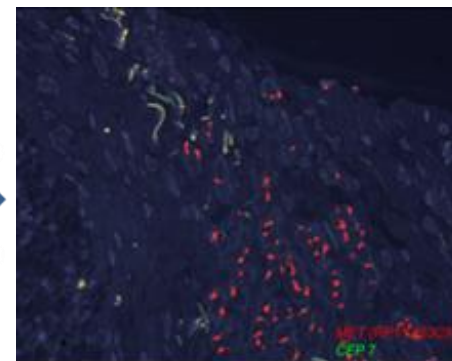

**Metastatic  
Lymph  
Node**

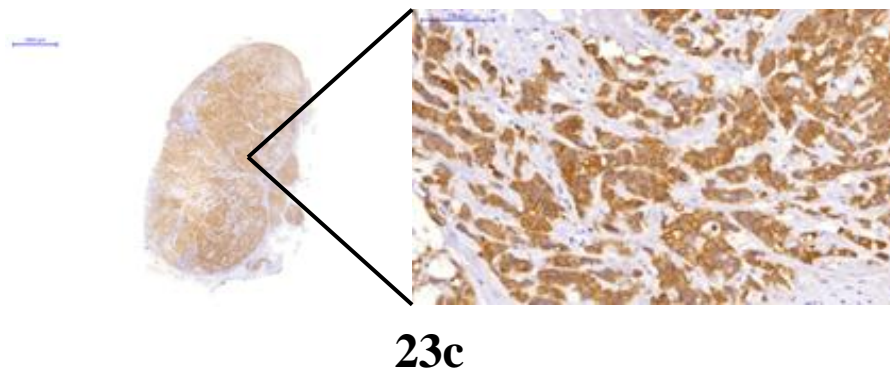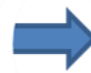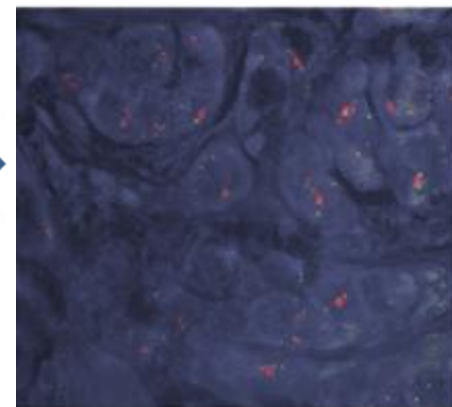

Supplement: Figure S2 — Correlation of IHC Met levels in the heterogeneous tissue sample #23 with MET FISH. The primary tumor (top row) was comprised of 75% IHC Met negative cells (GEC23b) and 25% Met positive (GEC23a), which correlated with FISH gene copy number (insets). The metastatic lymph node (bottom row, 23c) showed Met expression in 100% of cells, and tumor cells were all amplified by MET/CEP7 ratio >2 (Met IHC low and high power). (see Table S3 for FISH scores and Met SRM values.). (PDF) [file pone.0100586.s002.pdf]

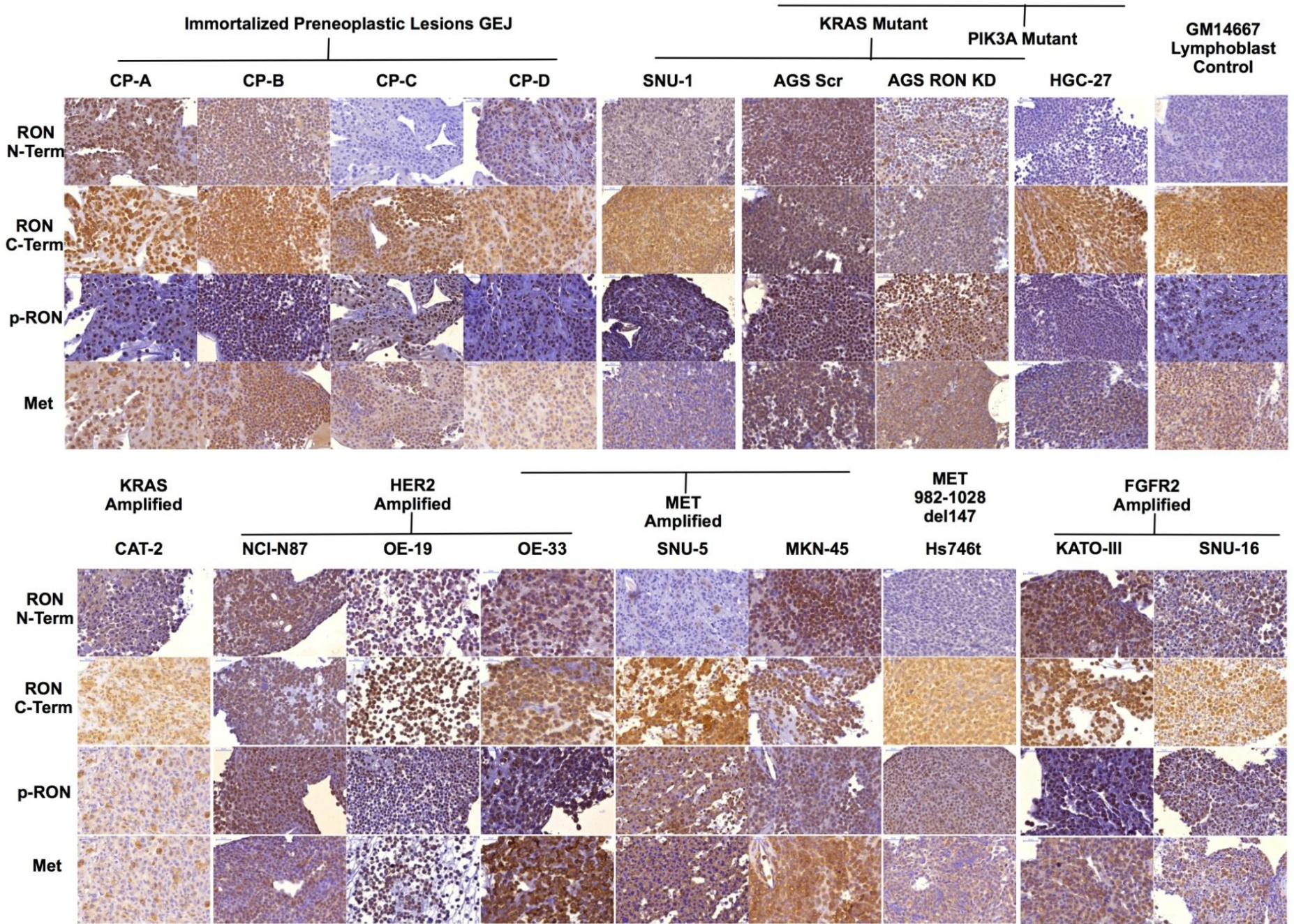

Supplementary Figure 3

Supplement: Figure S3 — Met IHC (bottom rows) for gastroesophageal cancer (GEC) cell line paraffin embedded pellets. For MET amplified lines (OE-33, SNU-5, and MKN-45) the difference of Met expression with non-amplified lines is under-appreciated by IHC, in contrast to levels observed with Mass Spectrometry (SRM-MS) (Table S2, Figure S1). RON tyrosine kinase, the other member in the MET tyrosine kinase family, is also demontrated with N-terminal, C-terminal, and phospho-RON (p-RON) antibody expression. Genomic characteristics of cell lines are represented above each cell line; GEJ, gastroesophageal junction. (PDF) [file pone.0100586.s003.pdf]

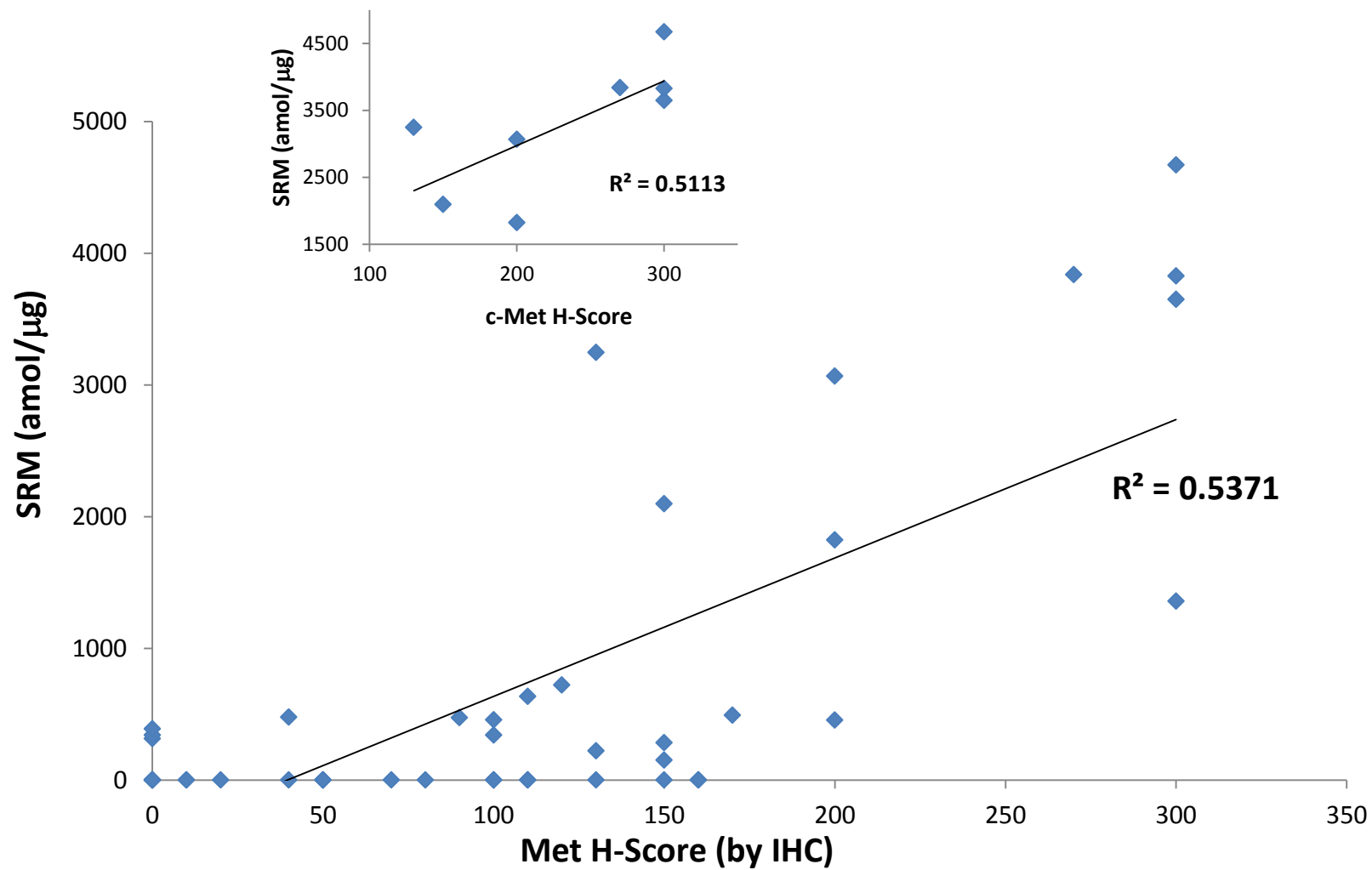

Supplement: Figure S4 — Correlation of Met levels using Liquid Tissue-SRM and Met H-score by IHC in 44 GEC tumors. Inset: comparison of SRM and Met H-score in 8 GEC tumors where Met expression level ≥1500 amol/mg. (PDF) [file pone.0100586.s004.pdf]

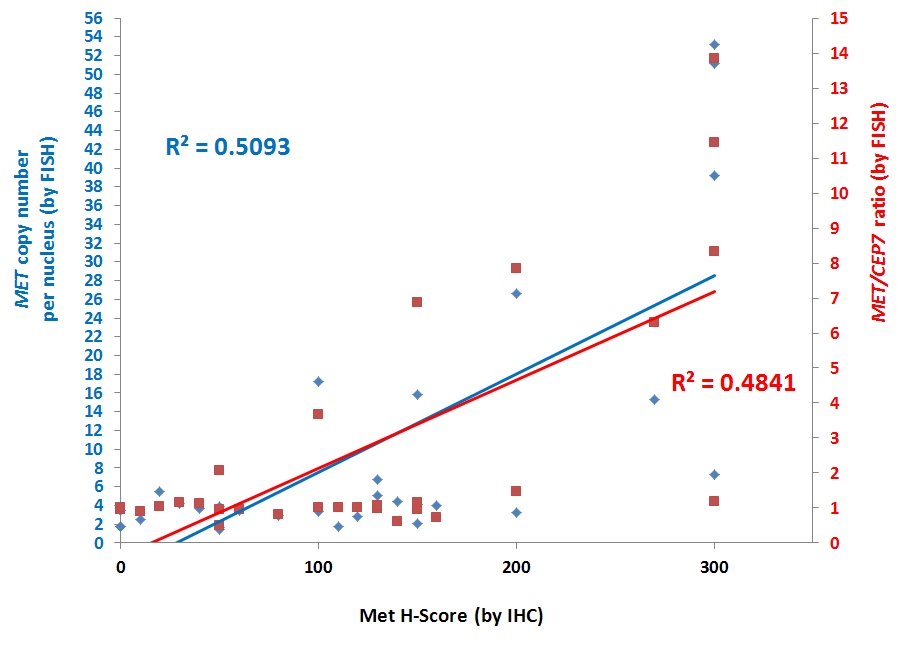

Supplement: Figure S5 — Correlation of Met H-score and MET gene amplification by FISH in 31 GEC tumors. The left y-axis (blue diamond) represents the MET copy number per nucleus and the right y-axis (red square) indicates MET:CEP7 ratio. (TIF) [file pone.0100586.s005.tif]
