# Supplementary material for: Absolute Quantitation of Met Using Mass Spectrometry for Clinical Application: Assay Precision, Stability, and Correlation with MET Gene Amplification in FFPE Tumor Tissue
Source: PLoS One. 2014 Jul 1;9(7):e100586. doi: 10.1371/journal.pone.0100586 (PMC4077664; doi:10.1371/journal.pone.0100586)
Supplement: Table S1 — Levels of SRM Met observed in 130 gastroesophageal cancer FFPE tissues. (DOCX) [file pone.0100586.s006.docx]

**Table S1**. Levels of SRM Met observed in 130 gastroesophageal cancer FFPE tissues.

| **Incoming Sample ID** | **Unique Sample Identifier** | **[cMet] (amol/μg total protein)** | **SD** | **CV** |
| --- | --- | --- | --- | --- |
| GEC1 | B0023-T2LR-A | ND |  |  |
| GEC3 | B0025-T1LR-A | positive |  |  |
| GEC5 | B0027-T2LR-A | 633.83 | 39.52 | 6.23 |
| GEC7 | B0029-T2LR-A | ND |  |  |
| GEC9 | B0031-T2LR-A | 339.98 | 32.80 | 9.65 |
| GEC11 | B0033-T2LR-A | ND |  |  |
| GEC12 | B0034-T2LR-A | ND |  |  |
| GEC13 | B0035-T2LR-A | ND |  |  |
| GEC14 | B0036-T2LR-A | ND |  |  |
| GEC15 | B0037-T2LR-A | ND |  |  |
| GEC16 | B0038-T2LR-A | ND |  |  |
| GEC17 | B0039-T2LR-A | ND |  |  |
| GEC18 | B0040-T2LR-A | ND |  |  |
| GEC19 | B0041-T2LR-A | 477.35 | 5.67 | 1.19 |
| GEC20 | B0042-T2LR-A | ND |  |  |
| GEC21 | B0043-T1LR-A | ND |  |  |
| GEC22 | B0044-T2LR-A | 313.30 | 41.86 | 13.36 |
| GEC23 | B0045-T2LR-A | 221.38 | 6.54 | 2.95 |
| GEC24 | B0046-T2LR-A | ND |  |  |
| GEC25 | B0047-T1LR-A | ND |  |  |
| GEC26 | B0595-T1LR-A | ND |  |  |
| GEC27 | B0596-P1R-A | ND |  |  |
| GEC28 | B0597-P1R-A | 3067.33 | 36.41 | 1.19 |
| GEC29 | B0598-P1R-A | 284.13 | 30.65 | 10.79 |
| GEC30 | B0599-P1R-A | ND |  |  |
| GEC31 | B0600-T1LR-A | ND |  |  |
| GEC32 | B0601-P1R-A | 491.37 | 35.05 | 7.13 |
| GEC33 | B0602-P1R-A | ND |  |  |
| GEC34 | B0603-P1R-A | ND |  |  |
| GEC35 | B0604-P1R-A | ND |  |  |
| GEC36 | B0605-T1LR-A | ND |  |  |
| GEC37 | B0606-P1R-A | ND |  |  |
| GEC39 | B0608-P1R-A | 473.90 | 80.04 | 16.89 |
| GEC40 | B0609-P1R-A | ND |  |  |
| GEC41 | B0610-P1R-A | ND |  |  |
| GEC42 | B0611-P1R-A | ND |  |  |
| GEC43 | B0612-P1R-A | ND |  |  |
| GEC44 | B0613-T1LR-A | 456.32 | 23.87 | 5.23 |
| GEC45 | B0614-P1R-A | 387.53 | 51.94 | 13.40 |
| GEC46 | B0615-P1R-A | 2097.83 | 46.26 | 2.21 |
| GEC47 | B0616-T1LR-A | ND |  |  |
| GEC48 | B0617-P1R-A | ND |  |  |
| GEC49 | B0618-P1R-A | ND |  |  |
| GEC50 | B0619-P1R-A | ND |  |  |
| GEC51 | B0620-P1R-A | ND |  |  |
| GEC52 | B0621-P1R-A | ND |  |  |
| GEC53 | B0622-P1R-A | ND |  |  |
| GEC54 | B0623-P1R-A | ND |  |  |
| GEC55 | B0624-P1R-A | ND |  |  |
| GEC56 | B0625-P1R-A | positive |  |  |
| GEC57 | B0626-P1R-A | ND |  |  |
| GEC58 | B0627-T1LR-A | ND |  |  |
| GEC59 | B0628-P1R-A | ND |  |  |
| GEC60 | B0629-P1R-A | ND |  |  |
| GEC62 | B0632-T1LR-A | ND |  |  |
| GEC64* | B0633-T1LR-A | 526.93* | 42.53 | 8.07 |
| GEC69 | B0636-T1LR-A | 341.17 | 37.60 | 11.02 |
| GEC70 | B0637-T1LR-A | ND |  |  |
| GEC71 | B0638-T1LR-A | 215.88 | 11.95 | 5.53 |
| GEC72 | B0639-T1LR-A | 1358.33 | 21.43 | 1.58 |
| GEC73 | B0640-T1LR-A | ND |  |  |
| GEC74 | B0641-T1LR-A | 332.97 | 54.71 | 16.43 |
| GEC75 | B0642-T1LR-A | 342.47 | 4.94 | 1.44 |
| GEC76 | B0643-T1LR-A | 360.55 | 8.91 | 2.47 |
| GEC77 | B0644-P1R-A | ND |  |  |
| GEC78 | B0645-T1LR-A | ND |  |  |
| GEC79 | B0646-P1R-A | ND |  |  |
| GEC80 | B0647-P1R-A | ND |  |  |
| GEC81 | B0648-T1LR-A | ND |  |  |
| GEC83 | B0650-T1LR-A | ND |  |  |
| GEC84 | B0651-P1R-A | ND |  |  |
| GEC85 | B0652-P1R-A | ND |  |  |
| GEC86 | B0653-P1R-A | ND |  |  |
| GEC87 | B0654-T1LR-A | ND |  |  |
| GEC88 | B0655-T1LR-A | ND |  |  |
| GEC89 | B0656-P1R-A | ND |  |  |
| GEC90 | B0657-T1LR-A | ND |  |  |
| GEC91 | B0658-P1R-A | ND |  |  |
| GEC92 | B0659-T1LR-A | 316.50 | 17.47 | 5.52 |
| GEC93 | B0660-P1R-A | 442.40 | 28.64 | 6.47 |
| GEC94 | B0661-P1R-A | ND |  |  |
| GEC95 | B0662-T1LR-A | ND |  |  |
| GEC96 | B0663-P1R-A | 444.63 | 3.08 | 0.69 |
| GEC97 | B0664-P1R-A | ND |  |  |
| GEC99 | B0666-T1LR-A | ND |  |  |
| GEC100 | B0667-P1R-A | ND |  |  |
| GEC102 | B0669-T1LR-A | ND |  |  |
| GEC103 | B0670-P1R-A | ND |  |  |
| GEC104 | B0671-T1LR-A | 720.67 | 68.39 | 9.49 |
| GEC106 | B0673-P1R-A | ND |  |  |
| GEC107 | B0674-P1R-A | ND |  |  |
| GEC113 | C0002-T1LR-A | 2369.50 | 81.7 | 3.45 |
| GEC114 | C0003-T1LR-A | 590.17 | 25.3 | 4.29 |
| GEC115 | C0004-T1LR-A | ND |  |  |
| GEC116 | C0005-T1LR-A | ND |  |  |
| GEC120 | C0007-T1LR-A | 4669.50 | 167.5 | 3.59 |
| GEC120b | C0008-T1LR-A | 3648.50 | 58.7 | 1.61 |
| GEC118 | C0173-P1R-A | 303.70 | 36.10 | 11.89 |
| GEC120c | C0174-P1R-A | 3827.33 | 115.54 | 3.02 |
| GEC121b | C0175-T1LR-A | ND |  |  |
| GEC127 | C0665-T1LR-A | ND |  |  |
| GEC147 | C0486-T1LR-A | 327.13 | 11.13 | 3.40 |
| LN/met of GEC5 S07-658-D1 | C0487-T1LR-A | 3836.83 | 58.79 | 1.53 |
| 2209371 | CC0101-T1LR-B | 286.20 | 1.48 | 0.52 |
| 2436501 | CC0102-T2LR-A | ND |  |  |
| 3233067 | CC0103-T2LR-A | 469.47 | 51.11 | 10.89 |
| 3301139 | CC0105-T1LR-A | 633.96 | 4.84 | 0.76 |
| 3310899 | CC0107-T1LR-A | 354.65 | 9.97 | 2.81 |
| NA | CC0108-P1R-B | ND |  |  |
| NA | CC0113-T1LR-A | ND |  |  |
| NA | CC0114-P1R-B | ND |  |  |
| NA | CC0115-P1R-B | ND |  |  |
| NA | CC0116-T1LR-B | ND |  |  |
| NA | CC0118-P1R-A | ND |  |  |
| NA | CC0119-P1R-A | ND |  |  |
| NA | CC0120-P1R-A | ND |  |  |
| NA | CC0121-T1LR-A | positive |  |  |
| NA | CC0122-P1R-B | 339.98 | 42.04 | 12.36 |
| NA | CC0123-T1LR-B | ND |  |  |
| NA | CC0126-T1LR-B | 662.33 | 17.75 | 2.68 |
| NA | CC0129-T1LR-B | 327.18 | 30.87 | 9.43 |
| NA | CC0133-T1LR-B | 870.50 | 24.56 | 2.82 |
| NA | CC0135-T1LR-B | 350.40 | 23.17 | 6.61 |
| NA | CC0136-T1LR-A | positive |  |  |
| NA | CC0137-T1LR-B | 339.55 | 2.33 | 0.69 |
| NA | CC0138-T1LR-B | 288.60 | 47.52 | 16.46 |
| NA | CC0139-T1LR-B | 362.92 | 36.80 | 10.14 |
| NA | CC0140-T1LR-A | ND |  |  |
| NA | CC0147-T1LR-B | 292.55 | 41.55 | 14.20 |
| NA | CC0151-T1LR-B | 378.83 | 23.51 | 6.21 |

**Legend:** SRM, selected reaction monitoring; ND, not detected; SD, standard deviation; CV, coefficient of variation. *sample from case in reference 18.
