# Supplementary material for: Absolute Quantitation of Met Using Mass Spectrometry for Clinical Application: Assay Precision, Stability, and Correlation with MET Gene Amplification in FFPE Tumor Tissue
Source: PLoS One. 2014 Jul 1;9(7):e100586. doi: 10.1371/journal.pone.0100586 (PMC4077664; doi:10.1371/journal.pone.0100586)
Supplement: Table S2 — Met protein level detected by SRM and MET GNC detected by FISH in GEC cell lines. (DOCX) [file pone.0100586.s007.docx]

**Table S2.** Met protein level detected by SRM and *MET* GNC detected by FISH in GEC cell lines.

| **Sample**  **No.** | **Cell Line** | **SRM** | **FISH** | | |
| --- | --- | --- | --- | --- | --- |
|  |  | **cMet (amol/μg total protein** | ***MET* GCN** | ***CEP7* GCN** | ***MET/CEP7*** |
| 1 | CP-A | 1254.33 | 3.28 | 3.23 | 1.02 |
| 2 | CP-B | ND | 2.34 | 2.16 | 1.08 |
| 3 | CP-C | 653.00 | 2.91 | 2.86 | 1.02 |
| 4 | CP-D | 915.50 | 7.13 | 3.96 | 1.8 |
| 5 | SNU-1 | ND | 2.92 | 3.27 | 0.89 |
| 6 | AGS | 347.68 | 2.08 | 2.05 | 1.01 |
| 7 | CAT-2 | 355.63 | 3.6 | 3.36 | 1.07 |
| 8 | CAT2R2 | 540.3 | 4.47 | 4.23 | 1.06 |
| 9 | CAT2R3 | 375.52 | 4.53 | 4.36 | 1.04 |
| 10 | CAT2R3as | 1001.25 | 4.33 | 4.12 | 1.05 |
| 11 | CAT3 | 331.4 | 3.96 | 4.04 | 0.98 |
| 12 | CAT4 | ND | 2.1 | 2.08 | 1.01 |
| 13 | HGC-27 | ND | 2.99 | 3.96 | 0.76 |
| 14 | MKN-1 | ND | 3.23 | 3.44 | 0.94 |
| 15 | NCI-N87 | 433.85 | 1.13 | 2.15 | 0.53 |
| 16 | OE19 | ND | 3.04 | 2.95 | 1.03 |
| **17** | **OE33** | **4463.33** | **36.00** | **6.73** | **5.35** |
| **18** | **SNU-5** | **6290.00** | **26.14** | **6.11** | **4.28** |
| **19** | **MKN-45** | **2774.00** | **25.73** | **5.7** | **4.51** |
| 20 | Hs746t | 150.00 | 3.52 | 2.81 | 1.25 |
| 21 | KATOIII | 1320.00 | 4.6 | 5.66 | 0.81 |
| 22 | SNU-16 | 507.55 | 4.9 | 4.97 | 0.99 |
| 23 | GM14667* | ND | 2.04 | 2.04 | 1.00 |

**Legend:** SRM, selected reaction monitoring; ND, Not Detected; GCN, Gene copy number; GEC, gastroesophageal cancer. *MET* FISH amplified (*MET/CEPT7* ratio > 2 and GCN > 4) cell lines are bolded.

*Lymphoblast control line: GM14667.
