# Supplementary material for: Absolute Quantitation of Met Using Mass Spectrometry for Clinical Application: Assay Precision, Stability, and Correlation with MET Gene Amplification in FFPE Tumor Tissue
Source: PLoS One. 2014 Jul 1;9(7):e100586. doi: 10.1371/journal.pone.0100586 (PMC4077664; doi:10.1371/journal.pone.0100586)
Supplement: Table S3 — Met protein level detected by SRM and MET GNC detected by FISH in 30 GEC FFPE tissues. (DOCX) [file pone.0100586.s008.docx]

**Table S3.** Met protein level detected by SRM and *MET* GNC detected by FISH in 30 GEC FFPE tissues.

| **Sample**  **No.** | **SRM** | **FISH** | | |
| --- | --- | --- | --- | --- |
|  | **cMet (amol/μg total protein** | ***MET* GCN** | ***CEP7* GCN** | ***MET/CEP7*** |
| 1 | ND | 1.65 | 3.32 | 0.50 |
| 2 | ND | 4.05 | 5.60 | 0.72 |
| 3 | ND | 2.9 | 3.5 | 0.80 |
| 4 | ND | 2.51 | 2.76 | 0.91 |
| 5 | ND | 1.49 | 1.60 | 0.93 |
| 6 | ND | 1.80 | 1.90 | 0.95 |
| 7 | ND | 1.49 | 1.56 | 0.96 |
| 8 | ND | 2.03 | 2.11 | 0.96 |
| 9 | ND | 1.72 | 1.80 | 0.96 |
| 10 | ND | 1.75 | 1.78 | 0.98 |
| 11 | ND | 1.68 | 1.72 | 0.98 |
| 12 | ND | 5.05 | 5.16 | 0.98 |
| 13 | ND | 2.20 | 2.20 | 1.00 |
| 14 | ND | 3.51 | 3.47 | 1.01 |
| 15 | ND | 5.53 | 5.33 | 1.04 |
| 16 | ND | 3.68 | 3.29 | 1.12 |
| 17 | ND | 3.89 | 1.89 | 2.06** |
| 18 | 150.00 | 4.10 | 3.55 | 1.15 |
| 19 | 316.50 | 3.80 | 2.90 | 1.30 |
| 20 | 341.17 | 3.40 | 3.40 | 1.00 |
| 21^ρ^ | 526.93 | 3.18 | 2.99 | 1.06 |
| 22 | 720.67 | 2.85 | 2.80 | 1.02 |
| **23a*** | **727.33** | **41.80** | **2.80** | **14.93** |
| 23b* | 727.33 | 3.74 | 3.62 | 1.03 |
| 24 | 1358.33 | 7.35 | 6.25 | 1.18 |
| **25** | **2097.83** | **15.80** | **2.30** | **6.87** |
| **26** | **2369.50** | **19.65** | **3.05** | **6.44** |
| **27** | **3067.33** | **26.65** | **3.40** | **7.84** |
| **28** | **3648.50** | **53.15** | **4.65** | **11.43** |
| **29** | **3827.33** | **39.20** | **4.70** | **8.34** |
| **23c*** | **3836** | **15.30** | **2.43** | **6.30** |
| **30** | **4669.50** | **51.20** | **3.70** | **13.84** |

**Legend:** ND, Not Detected; GCN, Gene copy number. *MET* amplified tumors are bolded.

^ρ^ This sample is taken from reference 18 (sample obtained at disease recurrence after initial onartuzumab treatment)

*Sample 23 showed MET cluster gene amplification (41.8 GNC/nuclues) in ~20-30% of tumors cells (23a). The remaining non-amplified tumor cells had low-polysomy (23b). SRM-Met (727.33 amol/μg) reflects both components weighted as percentage of tumor nuclei present. Sample 23c represents tumor infiltrated metastatic lymph node, where all tumor nuclei were gene cluster amplified with high Met expression (3836 amol/μg).

** This sample was considered NOT amplified despite ratio >2, due to loss of copy of *CEP7*, and *MET* GCN < 4.
