# Supplementary material for: Absolute Quantitation of Met Using Mass Spectrometry for Clinical Application: Assay Precision, Stability, and Correlation with MET Gene Amplification in FFPE Tumor Tissue
Source: PLoS One. 2014 Jul 1;9(7):e100586. doi: 10.1371/journal.pone.0100586 (PMC4077664; doi:10.1371/journal.pone.0100586)
Supplement: Table S4 — Met expression by IHC and SRM in 44 GEC FFPE tissues. (DOCX) [file pone.0100586.s009.docx]

| **Table S4.** Met expression by IHC and SRM in 44 GEC FFPE tissues. | | | | | | | | | | | | |
| --- | --- | --- | --- | --- | --- | --- | --- | --- | --- | --- | --- | --- |
|  | **IHC** | | | | | | | | | | | **SRM** |
| **ID** | **Score**  **0** | **% positive** | **Score**  **1** | **% positive** | **Score**  **2** | **% positive** | **Score**  **3** | **% positive** | **Score (>25%)** | **Score (>50%)** | **H-Score** | **Met**  **(amol/μg)** |
| 1 | 0 | 100 |  |  |  |  |  |  | N | N | 0 | ND |
| 2 | 0 | 100 |  |  |  |  |  |  | N | N | 0 | ND |
| 3 | 0 | 100 |  |  |  |  |  |  | N | N | 0 | ND |
| 4 | 0 | 100 |  |  |  |  |  |  | N | N | 0 | ND |
| 5 | 0 | 100 |  |  |  |  |  |  | N | N | 0 | 313.3 |
| 6 | 0 | 100 |  |  |  |  |  |  | N | N | 0 | 339.98 |
| 7 | 0 | 100 |  |  |  |  |  |  | N | N | 0 | 387.53 |
| 8 | 0 | 90 | 1 | 10 |  |  |  |  | N | N | 10 | ND |
| 9 | 0 | 90 | 1 | 10 |  |  |  |  | N | N | 10 | ND |
| 10 | 0 | 80 | 1 | 20 |  |  |  |  | N | N | 20 | ND |
| 11 | 0 | 60 | 1 | 40 |  |  |  |  | P | N | 40 | ND |
| 12 | 0 | 80 | 1 | 10 |  |  | 3 | 10 | N | N | 40 | 477.35 |
| 13 | 0 | 70 | 1 | 10 | 2 | 20 |  |  | P | N | 50 | ND |
| 14 | 0 | 50 | 1 | 50 |  |  |  |  | P | P | 50 | ND |
| 15 | 0 | 50 | 1 | 50 |  |  |  |  | P | P | 50 | ND |
| 16 | 0 | 30 | 1 | 70 |  |  |  |  | P | P | 70 | ND |
| 17 | 0 | 50 | 1 | 20 | 2 | 30 |  |  | P | P | 80 | ND |
| 18 | 0 | 30 | 1 | 50 | 2 | 20 |  |  | P | P | 90 | 473.9 |
| 19 |  |  | 1 | 100 |  |  |  |  | P | P | 100 | ND |
| 20 |  |  | 1 | 100 |  |  |  |  | P | P | 100 | ND |
| 21 |  |  | 1 | 100 |  |  |  |  | P | P | 100 | 341.17 |
| 22 |  |  | 1 | 100 |  |  |  |  | P | P | 100 | 456.32 |
| 23 | 0 | 10 | 1 | 70 | 2 | 20 |  |  | P | P | 110 | ND |
| 24 | 0 | 30 | 1 | 30 | 2 | 40 |  |  | P | P | 110 | ND |
| 25 | 0 | 60 | 1 | 5 |  |  | 3 | 35 | P | N | 110 | 633.83 |
| 26 |  |  | 1 | 80 | 2 | 20 |  |  | P | P | 120 | 720.67 |
| 27 | 0 | 20 | 1 | 30 | 2 | 50 |  |  | P | P | 130 | ND |
| 28 | 0 | 20 | 1 | 30 | 2 | 50 |  |  | P | P | 130 | 221.38 |
| 29 | 0 | 10 | 1 | 50 | 2 | 40 |  |  | P | P | 130 | **3245.5** |
| 30 | 0 | 10 | 1 | 30 | 2 | 60 |  |  | P | P | 150 | ND |
| 31 | 0 | 20 | 1 | 10 | 2 | 70 |  |  | P | P | 150 | 150 |
| 32 |  |  | 1 | 50 | 2 | 50 |  |  | P | P | 150 | 284.13 |
| 33 | 0 | 50 |  |  |  |  | 3 | 50 | P | P | 150 | **2097.83** |
| 34 | 0 | 10 | 1 | 20 | 2 | 70 |  |  | P | P | 160 | ND |
| 35 | 0 | 5 | 1 | 30 | 2 | 65 |  |  | P | P | 160 | ND |
| 36 | 0 | 10 | 1 | 10 | 2 | 80 |  |  | P | P | 170 | 491.37 |
| 37 |  |  |  |  | 2 | 100 |  |  | P | P | 200 | 455.0 |
| 38 | 0 | 20 | 1 | 20 |  |  | 3 | 60 | P | P | 200 | **1823.17** |
| 39 |  |  |  |  | 2 | 100 |  |  | P | P | 200 | **3067.33** |
| 40 | 0 | 10 |  |  |  |  | 3 | 90 | P | P | 270 | **3836.83** |
| 41 |  |  |  |  |  |  | 3 | 100 | P | P | 300 | **1358.33** |
| 42 |  |  |  |  |  |  | 3 | 100 | P | P | 300 | **3648.5** |
| 43 |  |  |  |  |  |  | 3 | 100 | P | P | 300 | **3827.33** |
| 44 |  |  |  |  |  |  | 3 | 100 | P | P | 300 | **4669.5** |

**Legend:** GCN, Gene copy number; N, negative; P, positive. *MET* amplified tumors (FISH ratio >2 and GCN > 4; or SRM >1500 amol/μg) are bolded.
