# Supplementary material for: Absolute Quantitation of Met Using Mass Spectrometry for Clinical Application: Assay Precision, Stability, and Correlation with MET Gene Amplification in FFPE Tumor Tissue
Source: PLoS One. 2014 Jul 1;9(7):e100586. doi: 10.1371/journal.pone.0100586 (PMC4077664; doi:10.1371/journal.pone.0100586)
Supplement: Table S5 — Met expression by IHC and MET GCN by FISH in 31 GEC tissues. (DOCX) [file pone.0100586.s010.docx]

|  | | | | | | | | | | | | | | |
| --- | --- | --- | --- | --- | --- | --- | --- | --- | --- | --- | --- | --- | --- | --- |
|  | **IHC** | | | | | | | | | | | **FISH** | | |
| **ID** | **Score** | **% positive** | **Score** | **% positive** | **Score** | **% positive** | **Score** | **% positive** | **Score (>25%)** | **Score (>50%)** | **H-Score** | **MET**  **GCN** | **CEP**  **GCN** | **FISH Ratio** |
| 1 | 0 | 100 |  |  |  |  |  |  | N | N | 0 | 1.8 | 1.9 | 0.95 |
| 2 | 0 | 100 |  |  |  |  |  |  | N | N | 0 | 1.72 | 1.8 | 0.96 |
| 3 | 0 | 100 |  |  |  |  |  |  | N | N | 0 | 3.51 | 3.47 | 1.01 |
| 4 | 0 | 90 | 1 | 10 |  |  |  |  | N | N | 10 | 2.51 | 2.76 | 0.91 |
| 5 | 0 | 80 | 1 | 20 |  |  |  |  | N | N | 20 | 5.53 | 5.33 | 1.04 |
| 6 | 0 | 70 | 1 | 30 |  |  |  |  | P | N | 30 | 4.23 | 3.67 | 1.15 |
| 7 | 0 | 80 | 1 | 10 |  |  | 3 | 10 | N | N | 40 | 3.68 | 3.29 | 1.12 |
| 8 | 0 | 50 | 1 | 50 |  |  |  |  | P | P | 50 | 1.65 | 3.32 | 0.5 |
| 9 | 0 | 50 | 1 | 50 |  |  |  |  | P | P | 50 | 1.49 | 1.56 | 0.96 |
| 10 | 0 | 70 | 1 | 10 | 2 | 20 |  |  | P | N | 50 | 3.89 | 1.89 | 2.06** |
| 11 | 0 | 60 | 1 | 20 | 2 | 20 |  |  | P | N | 60 | 3.45 | 3.65 | 0.95 |
| 12 | 0 | 50 | 1 | 20 | 2 | 30 |  |  | P | P | 80 | 2.9 | 3.5 | 0.8 |
| 13 |  |  | 1 | 100 |  |  |  |  | P | P | 100 | 3.4 | 3.4 | 1 |
| 14 |  |  | 1 | 100 |  |  |  |  | P | P | 100 | **17.25** | **4.7** | **3.67** |
| 15 | 0 | 10 | 1 | 70 | 2 | 20 |  |  | P | P | 110 | 1.74 | 1.71 | 1.02 |
| 16 | 0 | 60 | 1 | 5 |  |  | 3 | 35 | P | N | 110 | **41.8** | **2.8** | **14.93** |
| 17 |  |  | 1 | 80 | 2 | 20 |  |  | P | P | 120 | 2.85 | 2.8 | 1.02 |
| 18 | 0 | 20 | 1 | 30 | 2 | 50 |  |  | P | P | 130 | 5.05 | 5.16 | 0.98 |
| 19 | 0 | 40 | 1 | 20 | 2 | 10 | 3 | 30 | P | P | 130 | 6.8 | 6.31 | 1.08 |
| 20 | 0 | 10 | 1 | 40 | 2 | 50 |  |  | P | P | 140 | 4.45 | 7.2 | 0.62 |
| 21 | 0 | 10 | 1 | 30 | 2 | 60 |  |  | P | P | 150 | 2.03 | 2.11 | 0.96 |
| 22 | 0 | 20 | 1 | 10 | 2 | 70 |  |  | P | P | 150 | 4.1 | 3.55 | 1.15 |
| 23 | 0 | 50 |  |  |  |  | 3 | 50 | P | P | 150 | **15.8** | **2.3** | **6.87** |
| 24 | 0 | 10 | 1 | 20 | 2 | 70 |  |  | P | P | 160 | 4.05 | 5.6 | 0.72 |
| 25 |  |  |  |  | 2 | 100 |  |  | P | P | 200 | 3.3 | 2.25 | 1.47 |
| 26 |  |  |  |  | 2 | 100 |  |  | P | P | 200 | **26.65** | **3.4** | **7.84** |
| 27 | 0 | 10 |  |  |  |  | 3 | 90 | P | P | 270 | **15.3** | **2.43** | **6.3** |
| 28 |  |  |  |  |  |  | 3 | 100 | P | P | 300 | 7.35 | 6.25 | 1.18 |
| 29 |  |  |  |  |  |  | 3 | 100 | P | P | 300 | **39.2** | **4.7** | **8.34** |
| 30 |  |  |  |  |  |  | 3 | 100 | P | P | 300 | **53.15** | **4.65** | **11.43** |
| 31 |  |  |  |  |  |  | 3 | 100 | P | P | 300 | **51.2** | **3.7** | **13.84** |

**Table S5.** Met expression by IHC and *MET* GCN by FISH in 31 GEC tissues.

**Legend:** GCN, Gene copy number; N, negative; P, positive. *MET* amplified tumors (FISH ratio >2) are bolded.

** This sample is considered NOT amplified despite ratio >2, due to loss of copy of *CEP7*, and *MET* GCN < 4.
