# Supplementary material for: Absolute Quantitation of Met Using Mass Spectrometry for Clinical Application: Assay Precision, Stability, and Correlation with MET Gene Amplification in FFPE Tumor Tissue
Source: PLoS One. 2014 Jul 1;9(7):e100586. doi: 10.1371/journal.pone.0100586 (PMC4077664; doi:10.1371/journal.pone.0100586)
Supplement: Table S6 — Met expression by IHC, SRM and MET GCN by FISH in 24 GEC tissues. (DOCX) [file pone.0100586.s011.docx]

**Table S6.** Met expression by IHC, SRM and *MET* GCN by FISH in 24 GEC tissues.

|  | **IHC** | | | **SRM** | **FISH** | | |
| --- | --- | --- | --- | --- | --- | --- | --- |
| **ID** | **Score (>25%)** | **Score (>50%)** | **H-Score** | **Met**  **(amol/μg)** | **MET**  **GCN** | **CEP7**  **GCN** | **FISH**  **Ratio** |
| 1 | N | N | 0 | 0 | 1.72 | 1.8 | 0.96 |
| 2 | N | N | 0 | 0 | 1.8 | 1.9 | 0.95 |
| 3 | N | N | 0 | 0 | 3.51 | 3.47 | 1.01 |
| 4 | N | N | 10 | 0 | 2.51 | 2.76 | 0.91 |
| 5 | N | N | 20 | 0 | 5.53 | 5.33 | 1.04 |
| 6 | N | N | 40 | 477.35 | 3.68 | 3.29 | 1.12 |
| 7 | P | P | 50 | 0 | 1.49 | 1.56 | 0.96 |
| 8 | P | P | 50 | 0 | 1.65 | 3.32 | 0.5 |
| 9 | P | N | 50 | 0 | 3.89 | 1.89 | 2.06** |
| 10 | P | P | 80 | 0 | 2.9 | 3.5 | 0.8 |
| 11 | P | P | 100 | 341.17 | 3.4 | 3.4 | 1 |
| 12 | P | P | 110 | 0 | 1.74 | 1.71 | 1.02 |
| 13 | P | P | 120 | 720.67 | 2.85 | 2.8 | 1.02 |
| 14 | P | P | 130 | 0 | 5.05 | 5.16 | 0.98 |
| 15 | P | P | 150 | 0 | 2.03 | 2.11 | 0.96 |
| 16 | P | P | 150 | 150 | 4.1 | 3.55 | 1.15 |
| 17 | P | P | 150 | 2097.83 | **15.8** | **2.3** | **6.87** |
| 18 | P | P | 160 | 0 | 4.05 | 5.6 | 0.72 |
| 19 | P | P | 200 | 3067.33 | **26.65** | **3.4** | **7.84** |
| 20 | P | P | 270 | 3836.83 | **15.3** | **2.43** | **6.3** |
| 21 | P | P | 300 | 1358.33 | 7.35 | 6.25 | 1.18 |
| 22 | P | P | 300 | 3827.33 | **39.2** | **4.7** | **8.34** |
| 23 | P | P | 300 | 4669.5 | **51.2** | **3.7** | **13.84** |
| 24 | P | P | 300 | 3648.5 | **53.15** | **4.65** | **11.43** |

**Legend:** GCN, Gene copy number; N, negative; P, positive. *MET* amplified tumors (FISH ratio >2) are bolded.

** This sample is considered NOT amplified despite ratio >2, due to loss of copy of *CEP7*, and *MET* GCN < 4.
